# Fluid restriction after pediatric cardiac surgery is independently associated With shorter mechanical ventilation: a retrospective cohort study With adjustment for cardiopulmonary bypass time

**DOI:** 10.3389/fped.2026.1860058

**Published:** 2026-08-11

**Authors:** Nicholas Richards, Joshua Davis, William V Stein, Adeeb Khan

**Affiliations:** 1 San Juan Regional Medical Center, Farmington, NM, United States; 2 Presbyterian Healthcare Services, Albuquerque, NM, United States; 3 University of Chicago, Chicago, IL, United States; 4 Case Western Reserve University, Cleveland, OH, United States

**Keywords:** acute kidney injury, cardiopulmonary bypass, congenital heart disease, fluid restriction, hypernatremia, mechanical ventilation, postoperative outcomes, STAT category

## Abstract

**Background:**

Cardiopulmonary bypass (CPB) for pediatric cardiac surgery triggers a systemic inflammatory response driving postoperative fluid retention, prolonged ventilation, and acute kidney injury (AKI). The Starship Hospital guideline provides a structured framework for postoperative fluid restriction, but North American observational validation adjusting for CPB time has been lacking.

**Objective:**

To evaluate the association between a Starship-derived fluid restriction protocol and clinical outcomes, with adjustment for operative complexity.

**Methods:**

Retrospective single-center cohort of 329 pediatric patients undergoing structural CHD surgery with cardiopulmonary bypass (April 2017–February 2025): 259 pre-protocol, 70 post-protocol. Non-CPB lateral thoracotomy cases were excluded *a priori*. Protocol parameters were embedded in the mandatory order set, substantially standardizing fluid prescribing. Outcomes were analyzed with linear and generalized linear models (gamma; negative binomial for ventilation days), adjusted for age, weight, and STAT category, with prespecified sensitivity analyses adding CPB time, post-CPB TEE ventricular function, and procedure year.

**Results:**

Mean CPB time was shorter post-protocol (83.0 vs. 101.2 min; *p* = 0.008); modified ultrafiltration was not used. Adjusted for age, weight, and STAT, post-protocol status was associated with 49% lower expected ventilation days (IRR: 0.51, 95% CI: 0.37–0.69; *p* < 0.001), 34% lower LOS (ratio: 0.66, 0.50–0.88; *p* = 0.005), and 28% lower furosemide exposure (ratio: 0.72, 0.64–0.83; *p* < 0.001). After adjustment for CPB time, the ventilation effect persisted (IRR: 0.63; *p* = 0.004); adding post-CPB TEE ventricular function, the effect remained significant (IRR: 0.69, 0.50–0.96; *p* = 0.026). AKI did not differ unadjusted (42.5% vs. 37.1%, *p* = 0.49) or adjusted. Postoperative hypernatremia (Na >145) fell from 78.7% to 62.9% (adjusted OR: 0.44, 0.22–0.89; *p* = 0.022).

**Conclusions:**

A Starship-derived fluid restriction protocol was independently associated with shorter mechanical ventilation, reduced furosemide exposure, and reduced hypernatremia, robust to full adjustment for operative covariates. AKI and LOS effects attenuated under full adjustment. Findings are hypothesis-generating and support proactive fluid restriction as a candidate intervention warranting prospective evaluation.

## Introduction

Congenital heart disease (CHD) is the most common congenital anomaly worldwide, affecting approximately 8 per 1,000 live births and frequently requiring surgical correction in infancy (1). The majority of these procedures are performed under cardiopulmonary bypass (CPB), which exposes patients to a well-characterized systemic inflammatory response that is central to the pathophysiology of postoperative fluid retention and organ dysfunction (2–4).

CPB initiates fluid overload through multiple interacting mechanisms. Blood contact with the artificial bypass circuit surface triggers activation of complement, coagulation, kallikrein, and fibrinolytic cascades (2). The resulting cytokine response, particularly involving IL-6, IL-8, and TNF-α, disrupts endothelial integrity and increases capillary permeability, causing plasma proteins and fluid to shift from the intravascular to the interstitial compartment (3, 4). Hemodilution from the crystalloid pump prime further reduces oncotic pressure and promotes fluid extravasation. Ischemia-reperfusion injury from aortic cross-clamping contributes additional oxidative stress and endothelial damage (2). In pediatric patients, these effects are amplified by the relatively larger extracorporeal circuit-to-patient size ratio, immature complement and immune systems in neonates and infants, and the greater susceptibility of developing organ systems to inflammatory injury (2). The result is predictable and often severe postoperative fluid overload, with reported incidence of clinically significant capillary leak syndrome exceeding 25% in pediatric CPB populations (3). Modified ultrafiltration (MUF) during or after CPB can partially attenuate this inflammatory fluid load but does not eliminate it, and not all centers utilize MUF protocols (5, 6).

CPB-induced fluid overload has been associated with AKI, prolonged mechanical ventilation, and extended ICU and hospital stay across multiple pediatric cohort studies and meta-analyses (7–11). A 2020 systematic review of 12 studies (*n* = 3,111 patients) reported a dose-dependent relationship between the degree of fluid overload and mortality, AKI, and prolonged stay (10). “Fluid creep,” defined as non-resuscitation IV fluid in excess of maintenance, has been separately identified as a modifiable contributor to postoperative fluid accumulation beyond what CPB itself generates (12). The existing evidence base is dominated by observational designs, and demonstrated relationships are associative rather than causal; randomized trials of comprehensive fluid restriction strategies in pediatric CHD surgery are lacking (9, 10, 13, 14).

The Starship Hospital clinical guideline (15) provided one of the first structured institutional frameworks for counteracting CPB-induced fluid retention through proactive restriction rather than reactive diuresis. By limiting maintenance fluids to 50% to 70% of standard rates, avoiding hypotonic solutions, and reserving diuretics for specific clinical indications, the Starship approach targets postoperative fluid accumulation at its source. The guideline was developed as an institutional consensus document and has not, to our knowledge, been prospectively validated in a randomized controlled trial. At the time of our protocol adoption in 2020, prospective validation in North American community hospital settings was absent, and practice remained highly variable internationally (16).

The Risk Adjustment in Congenital Heart Surgery (RACHS-1) system stratifies CHD procedures into six complexity tiers (17), and the Society of Thoracic Surgeons-European Association for Cardio-Thoracic Surgery (STAT) mortality categorization (18) provides complementary risk stratification by procedure type. CPB time and cross-clamp time are independent operative determinants of postoperative morbidity and have not been incorporated in prior observational reports of pediatric fluid restriction interventions (9–11, 14, 19). The Starship Hospital guideline has been the most widely cited structured framework for postoperative fluid restriction in pediatric cardiac surgery (15), but to our knowledge it has not previously been validated in a North American population, nor has any prior observational evaluation of pediatric postoperative fluid restriction adjusted for both CPB time and STAT category.

The present study addresses these gaps by evaluating the association between a Starship-derived fluid restriction protocol and clinical outcomes in 329 pediatric patients undergoing structural CHD surgery with cardiopulmonary bypass at a single United States center, with adjustment for age, weight, STAT mortality category, CPB time, and post-CPB intraoperative TEE ventricular function. Lateral thoracotomy cases without CPB (coarctation repair and PDA ligation) were excluded *a priori* because their postoperative fluid physiology differs fundamentally from that of CPB cases. This is, to our knowledge, the first North American validation of a Starship-derived pediatric postoperative fluid restriction protocol and the first observational evaluation in any setting to combine *a priori* CPB-cohort restriction with 100% operative-covariate completeness and order-set-anchored prescribing standardization. The primary outcome of mechanical ventilation duration was chosen because it represents the most direct clinical consequence of postoperative pulmonary fluid accumulation and because reductions in ventilator dependence carry substantial implications for sedation exposure, infection risk, and family experience.

## Methods

### Study design and population

This retrospective cohort study included all pediatric patients (<18 years) undergoing open surgical intervention for structural CHD with cardiopulmonary bypass between April 2017 and February 2025 at the Pediatric Intensive Care Unit (PICU) of Presbyterian Hospital, Albuquerque, New Mexico. IRB approval was obtained (Presbyterian Healthcare Services IRB Exempt, 45 CFR 46.104(d)(4)(iii); effective January 27, 2025). Patients were assigned to pre-protocol (April 2017 to April 2022; *n* = 259) or post-protocol (May 2022 to February 2025; *n* = 70) cohorts based on the date of the first qualifying procedure.

From an initial pool of 435 candidate cases identified by procedure code, two sequential exclusions were applied to produce the analytic cohort. First, 26 non-structural cases (perinatal cardiac events, device-related complications, and peripheral vascular procedures) were excluded based on prespecified ICD-10 codes (P29.30, I31.4, T82.857A, T82.858A, I31.3, I51.3, P29.0, P29.89, Q27.8). Second, 106 lateral thoracotomy cases that did not involve cardiopulmonary bypass (coarctation of the aorta repair and patent ductus arteriosus ligation) were excluded because their physiology and postoperative fluid management requirements differ fundamentally from those of CPB cases. Postoperative fluid retention in cardiac surgery is driven primarily by the CPB-induced systemic inflammatory response and consequent capillary leak, mechanisms that do not apply to non-CPB lateral thoracotomy. Including these cases would dilute the cohort with a physiologically different population and introduce a misleading source of variability into multivariable models. The resulting analytic cohort comprised 329 CPB cases, with CPB time and cross-clamp time available for 100% of analyzed patients.

Group assignment was determined by calendar time rather than randomization. This non-randomized before-and-after design carries an *a priori* risk of temporal confounding from concurrent changes in surgical, perfusion, anesthetic, and ICU practice. The risk of confounding is addressed analytically through a prespecified year-adjusted sensitivity analysis and qualitatively in the Discussion. The imbalance in group sizes (259 vs. 70) reflects the longer pre-protocol observation window (61 months vs. 34 months) and a lower mean annual surgical volume during the post-protocol era. Implications for statistical power, particularly for binary and subgroup analyses, are addressed in the Limitations.

### Perioperative management and care pathway standardization

Beyond the fluid management protocol itself, several elements of perioperative care evolved during the study period. The institution's pediatric cardiac surgical and perfusion teams were broadly stable. Documented practice changes included incremental adoption of miniaturized CPB circuits with reduced prime volumes, refinements in myocardial protection, increased use of point-of-care coagulation testing, and lung-protective ventilation strategies in the operating room and ICU. Modified ultrafiltration (MUF) was not used in any patient in either cohort during the study period, eliminating MUF as a potential confounder of the observed associations. Anesthetic agents, vasoactive infusion choices, sedation pathways, and nurse-to-patient ratios followed unit-level practice rather than a single written protocol. Other than the fluid management bundle introduced in May 2022, no additional formal care pathway was implemented or revised during the study period; informal practice drift cannot be excluded.

Cardiopulmonary bypass (CPB) time and aortic cross-clamp time (CCT) were retrieved from the operative record for 100% of the 329 analyzed CPB patients. Ventricular function was assessed by intraoperative transesophageal echocardiography (TEE) performed in the operating room after weaning from cardiopulmonary bypass and before transfer to the cardiac ICU; findings were abstracted from the operative TEE report and categorized as good or any degree of depression (mild, moderate, or severe). Because the post-CPB TEE assessment is performed in the operating room before any postoperative fluid management has occurred, post-CPB VF status is a purely intraoperative variable that reflects the operative course rather than any downstream protocol effect. It therefore belongs in the operative covariate set alongside CPB time and STAT category and is included as such in sensitivity analyses (Tables 1, 2). STAT mortality categories were derived from each patient's primary CHD diagnosis using the Society of Thoracic Surgeons-European Association for Cardio-Thoracic Surgery STAT classification (Jacobs et al., 2009). All 329 analyzed patients received a STAT category. Fully adjusted multivariable models incorporating CPB time, STAT category, and post-CPB intraoperative TEE ventricular function were performed in this complete-data cohort.

**Table 1:** Fully adjusted sensitivity analysis (CPB cohort, *n* = 329, +CPB time).

Outcome (model)	Effect estimate (post vs. pre)	95% CI	*p*-value
AKI (logistic)	0.87 (OR)	0.46–1.63	0.66
Hospital LOS (gamma, log link)	0.76	0.58–1.00	0.052
Ventilation days (negative binomial)	0.63 (IRR)	0.45–0.86	0.004
Furosemide mg/kg (gamma, log link)	0.75	0.65–0.85	<0.001
AKI, year-adjusted	0.29 (OR)	0.10–0.83	0.021
Hospital LOS, year-adjusted	0.39	0.25–0.60	<0.001
Ventilation days, year-adjusted	0.30 (IRR)	0.18–0.50	<0.001
Furosemide mg/kg, year-adjusted	0.97	0.77–1.21	0.77

All models adjusted for age, weight, CPB time, and STAT mortality category. Year-adjusted models additionally include procedure year. Analysis in the CPB-only cohort (*n* = 329, with CPB time available for 100% of analyzed patients; 259 pre-protocol, 70 post-protocol). Effect estimates reported as odds ratio (OR), ratio of geometric means (gamma models), or incidence rate ratio (IRR). CPB time was an independent predictor of AKI (OR: 1.01 per min; *p* < 0.001), LOS (ratio: 1.006 per min; *p* < 0.001), and ventilation days (IRR: 1.009 per min; *p* < 0.001) in all models.

**Table 2:** Post-CPB intraoperative TEE ventricular function: associations and sensitivity analyses.

Analysis	Reference/Group 1	Comparison/Group 2	Effect estimate	*p*
Any depression by cohort	Pre: 53/258 (20.5%)	Post: 5/70 (7.1%)	Fisher exact	0.008
VF depression → POD 1 IV input mL/kg	Good: 7.96 ± 7.39	Depressed: 13.92 ± 16.30	Mann–Whitney	0.005
VF depression → POD 2 IV input mL/kg	Good: 8.41 ± 9.04	Depressed: 17.78 ± 17.95	Mann–Whitney	<0.001
VF depression → AKI (unadj.)	Good: 94/270 (34.8%)	Depressed: 41/58 (70.7%)	RR: 2.03	<0.001
VF depression → AKI (adj. age/wt/STAT)	Good function	Any depression	OR: 3.01 (1.51–5.99)	0.002
VF depression → ventilation days	Good function	Any depression	IRR: 3.32 (2.43–4.55)	<0.001
VF depression → hospital LOS (gamma)	Good function	Any depression	ratio: 2.16 (1.58–2.94)	<0.001
Protocol → AKI (without VF)	Pre-protocol	Post-protocol	OR: 0.67 (0.37–1.23)	0.20
Protocol → AKI (+VF)	Pre-protocol	Post-protocol	OR: 0.80 (0.43–1.48)	0.48
Protocol → AKI (+VF + CPB time)	Pre-protocol	Post-protocol	OR: 0.91 (0.48–1.71)	0.77
Protocol → ventilation (without VF)	Pre-protocol	Post-protocol	IRR: 0.51 (0.37–0.69)	<0.001
Protocol → ventilation (+VF)	Pre-protocol	Post-protocol	IRR: 0.68 (0.49–0.93)	0.015
Protocol → ventilation (+VF + CPB time)	Pre-protocol	Post-protocol	IRR: 0.69 (0.50–0.96)	0.026

Ventricular function was assessed by intraoperative transesophageal echocardiography in the operating room after weaning from CPB, before transfer to the cardiac ICU. This timing makes post-CPB VF status a purely intraoperative observation that captures the operative trajectory rather than a downstream outcome of the protocol. *n* = 328 with VF available; *n* = 257 in models additionally adjusting for CPB time. The protocol-attributable ventilation effect persists under all adjustments including this operative-state covariate.

Twenty-six patients were excluded from the initial pool: 14 with pulmonary hypertension of the newborn (P29.30), 2 with cardiac tamponade (I31.4), 3 with cardiac prosthetic device stenosis (T82.857A), 2 with vascular prosthetic device stenosis (T82.858A), 1 with pericardial effusion (I31.3), 1 with intracardiac thrombosis (I51.3), 1 with neonatal cardiac failure (P29.0), 1 with other perinatal cardiovascular disorder (P29.89), and 1 with peripheral vascular malformation (Q27.8). These diagnoses represent perinatal physiologic disorders, device-related re-interventions, or non-structural cardiovascular conditions whose postoperative fluid physiology differs meaningfully from primary structural CHD repair. A further 106 patients undergoing lateral thoracotomy without cardiopulmonary bypass (coarctation of the aorta repair and patent ductus arteriosus ligation) were excluded because they do not experience CPB-induced systemic inflammatory capillary leak, the dominant driver of postoperative fluid retention that the protocol is designed to mitigate. The final analytic cohort comprised 329 patients undergoing structural CHD surgery with cardiopulmonary bypass.

### RACHS-1 classification

Each patient's primary CHD diagnosis was assigned a RACHS-1 category using the validated classification by Jenkins et al. (12). All 329 analyzed patients in the CPB cohort received RACHS-1 classification.

### CPB-Directed fluid restriction protocol

The protocol was adapted from the Starship Hospital guideline (Finucane and Hamer, 2016) (15). Core elements were as follows: (1) intravenous maintenance fluids restricted to 50% of standard rates on the day of bypass and 50% to 70% for the first 48 postoperative hours; (2) avoidance of hypotonic solutions; (3) delayed electrolyte supplementation until hemodynamic and renal stabilization; (4) diuretics reserved for specific clinical indications rather than prophylactic use; and (5) early transition to enteral nutrition beginning on postoperative Day 2 to reduce IV fluid requirements. Non-bypass procedures used a 70% maintenance restriction.

### Protocol adherence

In the post-protocol cohort, the fluid restriction parameters were embedded into the institutional electronic order set for the postoperative care of pediatric cardiac surgery patients. Use of the order set was mandatory for all postoperative admissions during the post-protocol period, and the restricted maintenance volume and avoidance of hypotonic fluid administration were therefore applied uniformly at the level of the prescribed order. Embedding the protocol into a mandatory order set is expected to have substantially improved standardization of maintenance fluid prescribing compared with voluntary clinician adherence. We acknowledge, however, that undocumented bedside deviations or individualized adjustments by clinicians at the point of care cannot be fully excluded in a retrospective analysis, and that order-set-based prescribing improves but does not absolutely guarantee real-world bedside implementation. Documented clinical deviations occurred when individualized fluid management was required (for example, active resuscitation, hemorrhage, or renal replacement therapy), and these were recorded in the standard medical record rather than tracked as protocol overrides because no formal override mechanism was needed under an order-set-anchored workflow. As an empirical confirmation of the order-set effect, postoperative IV maintenance fluid input on Day 1 was a median of 4.2% (mean 6.3%) of calculated Holliday-Segar maintenance in the post-protocol cohort vs. a median of 7.1% (mean 10.2%) in the pre-protocol cohort. These absolute IV input values are well below standard maintenance in both cohorts and reflect the protocol-restricted IV maintenance component rather than total daily fluid intake (which includes enteral nutrition, blood products, and medication carriers). The directionally consistent reduction across all five postoperative days (Table 3) is concordant with broad order-set-anchored adherence.

**Table 3:** Daily fluid input and output (mL/kg/day).

POD	Input Pre mL/kg	Input Post mL/kg	p	Output Pre mL/kg	Output Post mL/kg	*p*
1	8.73 ± 9.69	5.39 ± 6.32	<0.001	23.45 ± 22.10	28.81 ± 27.72	0.229
2	10.15 ± 11.76	5.69 ± 6.16	<0.001	33.49 ± 38.44	42.44 ± 37.15	0.047
3	7.75 ± 8.64	4.35 ± 6.24	<0.001	–	–	–
4	6.85 ± 8.17	3.61 ± 5.49	0.001	–	–	–
5	6.92 ± 9.10	3.41 ± 5.54	<0.001	–	–	–

Values are mean ± SD. Mann–Whitney *U* test. PO*D* = postoperative day. Output data available Days 1–2 only.

### Outcomes and data collection

Primary outcomes were total diuretic doses and furosemide mg/kg over the first seven postoperative days. Secondary outcomes included AKI, hospital LOS, ICU LOS, mechanical ventilation days, daily fluid input/output (mL/kg/day), serum electrolytes and metabolic markers (Days 1–3), percentage weight change, positive culture rate, and in-hospital mortality.

AKI was defined using KDIGO serum creatinine criteria, with baseline creatinine taken as the most recent preoperative value within 90 days. Urine output-based KDIGO criteria were not applied. Only daily total urine output was recorded in the retrospective dataset, and the hourly values required to identify the consecutive-hour windows specified by KDIGO (e.g., <0.5 mL/kg/h for 6 + consecutive hours for Stage 1) could not be extracted; daily totals were additionally missing for 37%–71% of patients depending on postoperative day, with missingness strongly correlated with patient age. A 24-h-average urine output rate computed from daily totals is also not interpretable as an AKI marker in this study because the fluid restriction protocol itself reduces total daily urine output by design through input restriction, independent of renal function. The methodological consequences and direction of bias are addressed in detail in the Limitations. The direction of misclassification is conservative: creatinine-only criteria are known to underdetect AKI relative to combined creatinine plus urine output criteria, biasing observed protocol-AKI associations toward the null.

### Statistical analysis

All primary variables had 0% missing data; laboratory variables had ≤8.5% missing. Missingness was assumed to be at random and was handled through complete-case analysis as the primary approach. To assess robustness, a prespecified multiple imputation sensitivity analysis was performed for laboratory outcomes using iterative chained-equations imputation (10 imputations, conditioned on protocol status, age, weight, CPB time, STAT, and the full lab covariate matrix); pooled estimates following Rubin's rules were compared with complete-case results (Table 4). Continuous outcomes are presented as mean ± SD with median; between-group comparisons used Mann–Whitney *U* tests. Standardized effect sizes (Cohen's *d* with 95% CI, Hedges’ *g*, and Cliff's delta) and risk differences with 95% CIs are reported for the primary clinical outcomes (Tables 4, 5) to facilitate cross-study comparison and to characterize effect magnitudes independent of statistical significance.

**Table 4:** Multiple imputation sensitivity analysis: postoperative sodium and hypernatremia (CPB cohort).

Variable	Pre CC (n)	Pre MI	Post CC (n)	Post MI	Method consistency
Max sodium POD 1 (mmol/L)	148.0 (246)	147.9	147.2 (68)	147.2	concordant
Max sodium POD 2 (mmol/L)	148.3 (250)	148.2	144.1 (70)	144.1	concordant
Max sodium POD 3 (mmol/L)	144.3 (249)	144.3	141.5 (70)	141.5	concordant
Avg sodium POD 1 (mmol/L)	147.0 (246)	146.9	146.0 (68)	146.0	concordant
Avg sodium POD 2 (mmol/L)	148.0 (250)	147.9	143.6 (70)	143.6	concordant
Hypernatremia (Na > 145) POD 1, %	70.7%	70.1%	61.8%	61.7%	concordant
Hypernatremia (Na > 145) POD 2, %	65.2%	64.7%	38.6%	38.6%	concordant
Hypernatremia (Na > 145) POD 3, %	32.9%	33.3%	21.4%	21.4%	concordant

CC, complete-case analysis; MI, multiple imputation (10 imputations using iterative chained equations, conditioned on protocol status, age, weight, CPB time, STAT category, and the full laboratory covariate matrix; pooled by Rubin's rules). Pre and Post columns report the mean (CC) or imputed mean (MI) within each cohort. Method consistency is defined as a between-method difference ≤0.5 mmol/L for sodium means or ≤1 percentage point for hypernatremia thresholds. All variables analyzed met this criterion, supporting the robustness of the complete-case results.

**Table 5:** Diuretic exposure: Pre- vs. Post-Protocol (CPB Cohort).

Outcome	Pre Mean ± SD [Median]	Post Mean ± SD [Median]	*p*	Cohen's *d* (95% CI)
Total diuretic doses	14.66 ± 8.32 [13]	10.40 ± 6.81 [10]	<0.001	0.53 (0.26 to 0.80)
Furosemide doses	11.54 ± 5.30 [11]	9.23 ± 4.75 [9.5]	0.002	0.45 (0.18 to 0.71)
Total furosemide, mg	107.39 ± 79.49 [92]	84.57 ± 61.04 [75.3]	0.033	0.30 (0.04 to 0.57)
Furosemide, mg/kg body weight	9.72 ± 6.18 [8.8]	7.46 ± 5.28 [7.1]	0.007	0.38 (0.11 to 0.64)
Spironolactone doses	0.89 ± 2.37 [0]	0.29 ± 1.21 [0]	0.029	0.28 (0.01 to 0.54)
Chlorothiazide doses	1.86 ± 3.04 [0]	0.77 ± 2.47 [0]	<0.001	0.37 (0.10 to 0.64)
Bumetanide doses	0.02 ± 0.28 [0]	0.03 ± 0.24 [0]	0.615	−0.02 (−0.28 to 0.24)
Unique diuretic agents per patient	1.67 ± 0.87 [1]	1.29 ± 0.66 [1]	<0.001	0.47 (0.20 to 0.73)

Values are mean ± SD [median]. Mann–Whitney *U* test. Cohen's *d* calculated with pooled standard deviation; positive d indicates pre > post (i.e., reduction with protocol). Hedges’ *g* values were nearly identical to *d* (within 0.01) for all outcomes given the cohort sizes and are not separately tabulated. Cliff's delta values ranged from 0.10 to 0.31, consistent with small-to-moderate rank-based effects. CPB-only cohort (*n* = 329).

Multivariable logistic regression evaluated independent associations with AKI (OR, 95% CI). For continuous outcomes (LOS, ventilation days, furosemide mg/kg), linear regression was performed for transparency and direct comparability with prior literature, and the linear coefficients are reported as the primary multivariable results. Given the right-skewed distributions of these outcomes (LOS skewness 2.79, ventilation days 4.71), generalized linear models with log link (gamma family for LOS and furosemide mg/kg; negative binomial for ventilation days) were performed as a prespecified sensitivity analysis, with effect estimates reported as ratios of geometric means or incidence rate ratios with 95% confidence intervals. All multivariable models adjusted for age, weight, and STAT category. Sensitivity analyses additionally adjusted for CPB time and post-CPB intraoperative TEE ventricular function. A separate prespecified sensitivity analysis additionally adjusted for procedure year as a continuous variable to address temporal confounding. All tests were two-sided, with significance defined at *p* < 0.05. Analyses were performed using Python 3.12 (statsmodels, scipy) and verified against the original SAS-based analyses; results were concordant.

**Figure 1:**
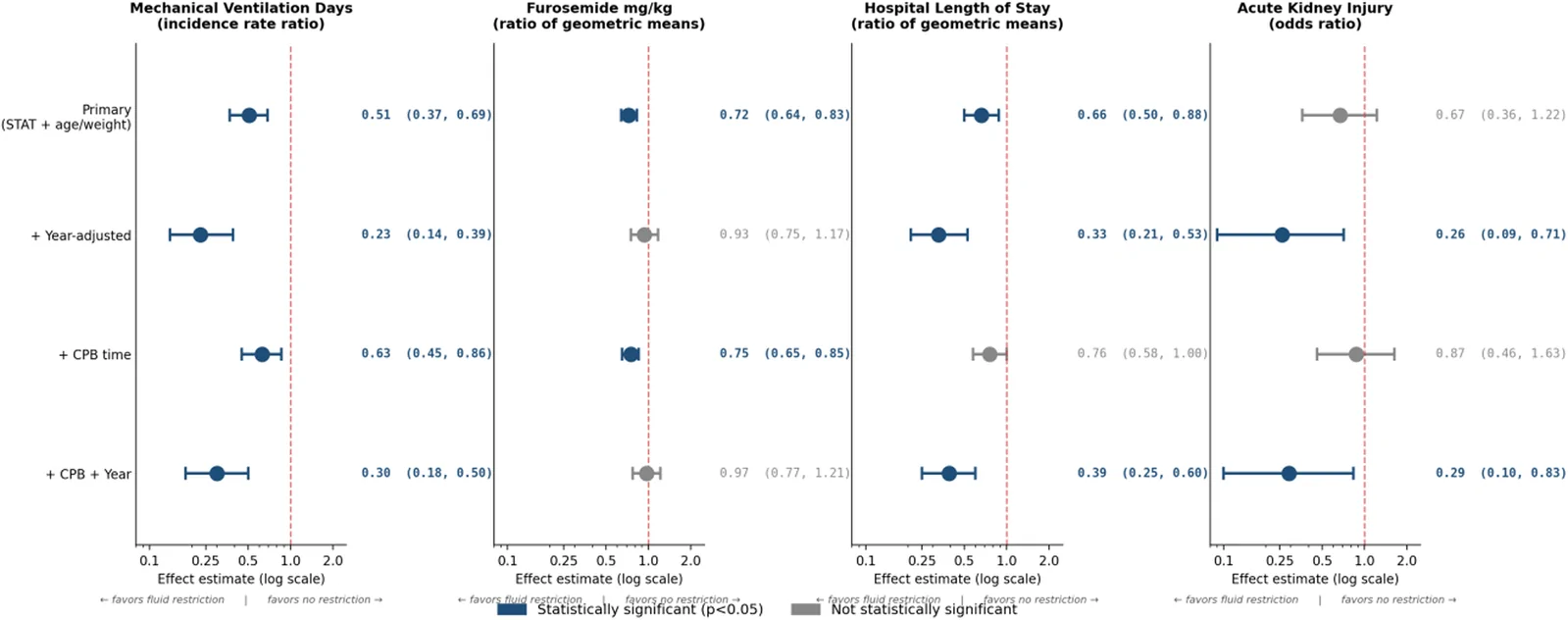
Adjusted effect estimates of fluid restriction protocol across multivariable models. Forest plot showing the post-protocol effect estimate (relative to pre-protocol) across four progressively adjusted multivariable models for each primary outcome in the CPB-only cohort (*n* = 329). Effect estimates: incidence rate ratio (ventilation days), ratio of geometric means (LOS, furosemide), odds ratio (AKI). All models adjusted for age, weight, and STAT category; year-adjusted models additionally adjusted for procedure year; fully-adjusted models additionally adjusted for CPB time. Estimates with 95% CI excluding 1.0 are shown in dark blue (statistically significant); others in gray.

To aid interpretation, analyses are designated as follows. The primary analyses are the comparisons of mechanical ventilation duration, hospital length of stay, furosemide exposure, and AKI between cohorts, with multivariable adjustment for age, weight, and STAT category (Tables 5–5d). All remaining analyses are designated as secondary or exploratory and are intended to characterize robustness and mechanism rather than to provide confirmatory tests: these include the log-transformed and year-adjusted sensitivity analyses (Table 6b), the fully-adjusted analyses incorporating CPB time and post-CPB intraoperative TEE ventricular function (Tables 1, 1), the STAT-stratified subgroup analysis (Table 7), the postoperative electrolyte and hypernatremia analyses (Tables 4, 8, 8b), the multiple imputation sensitivity analysis (Table 4), and the post-CPB ventricular function prognostic analyses (Table 2). Because no adjustment for multiple comparisons was applied, the secondary and exploratory results should be interpreted as hypothesis-generating, and *p*-values from these analyses should not be treated as confirmatory. Effect estimates for each primary outcome across all four adjustment levels are displayed together in Figure 1.

**Table 5b:** Clinical outcomes: Pre- vs. Post-Protocol (CPB Cohort).

Outcome	Pre Mean ± SD [Median]	Post Mean ± SD [Median]	*p*	Effect size (95% CI)
Hospital LOS, days	18.83 ± 24.93 [8]	12.33 ± 16.77 [6]	0.054	*d* = 0.28 (0.01 to 0.54)
ICU LOS, days	18.42 ± 24.59 [8]	12.27 ± 16.76 [6]	0.090	*d* = 0.27 (0.00 to 0.53)
Mechanical ventilation, days	7.12 ± 15.18 [2]	4.01 ± 9.10 [1]	<0.001	*d* = 0.22 (−0.04 to 0.48)
AKI incidence, *n* (%)	110 (42.5%)	26 (37.1%)	0.49	RD +5.3 pp (−7.5 to +18.1)

Values are mean ± SD [median] or *n* (%). CPB-only cohort (*n* = 329). Effect sizes: Cohen's *d* (pooled SD) for continuous outcomes; risk difference (RD, percentage points) for binary AKI outcome. Positive d and positive RD indicate pre > post (i.e., reduction with protocol). Hedges’ *g* and Cliff's delta values for continuous outcomes: LOS *g* = 0.28, Cliff's *δ* = 0.15; ICU LOS *g* = 0.26, Cliff's *δ* = 0.13; ventilation *g* = 0.22, Cliff's *δ* = 0.30 (the rank-based Cliff's *δ* is notably larger than d for ventilation days, consistent with the strong right-skew and the more robust rank-based GLM result in Table 6b). AKI defined by KDIGO serum creatinine criteria only; urine output criteria were not applied (see Methods, Limitations).

**Table 5c:** Primary multivariable analysis: independent predictors of AKI, LOS, and ventilation days (CPB cohort).

Variable	AKI OR	AKI 95% CI	AKI *p*	LOS *β* (days)	LOS 95% CI	LOS *p*
Post-protocol vs. pre	0.67	0.36–1.22	0.190	−7.34	−13.13 to −1.54	0.013
Age, years	0.68	0.57–0.82	<0.001	−1.72	−2.99 to −0.46	0.008
Weight, kg	1.04	1.00–1.09	0.059	0.05	−0.27 to 0.37	0.769
STAT category	1.42	1.00–2.01	0.047	5.32	1.99 to 8.64	0.002

Logistic regression (AKI): OR (95% CI). Linear regression (LOS): β (95% CI). Ventilation-days post-protocol coefficient: *β* = −3.48 (95% CI −7.08 to 0.12; *p* = 0.058). CPB-only cohort (*n* = 329). Covariates: post-protocol status, age, weight, STAT mortality category.

**Table 5d:** Sensitivity analysis: year-adjusted multivariable models (CPB cohort).

Variable	AKI OR	AKI 95% CI	AKI *p*	LOS β (days)	LOS 95% CI	LOS *p*
Post-protocol vs. pre	0.26	0.09–0.71	0.009	−19.57	−29.19 to −9.95	<0.001
Procedure year	1.26	1.03–1.52	0.022	2.89	1.06 to 4.72	0.002

All models additionally adjusted for age, weight, and STAT category. Furosemide mg/kg model: post-protocol *β* = +0.27 (95% CI: −1.85 to +2.38; *p* = 0.80); procedure year *β* = −0.64/year (*p* = 0.002). The full attenuation of the post-protocol furosemide coefficient when year is included indicates a strong parallel secular trend in diuretic use. CPB-only cohort (*n* = 329).

**Table 6b:** Log-Transformed sensitivity analysis (GLM with Log link, CPB cohort).

Outcome (model)	Effect estimate (post vs. pre)	95% CI	*p*-value
Hospital LOS (gamma, log link)	0.66	0.50–0.88	0.005
Ventilation days (negative binomial)	0.51 (IRR)	0.37–0.69	<0.001
Furosemide mg/kg (gamma, log link)	0.72	0.64–0.83	<0.001
Hospital LOS, year-adjusted	0.33	0.21–0.53	<0.001
Ventilation days, year-adjusted	0.23 (IRR)	0.14–0.39	<0.001
Furosemide mg/kg, year-adjusted	0.93	0.75–1.17	0.55

All models adjusted for age, weight, and STAT category. Effect estimates reported as ratio of geometric means (gamma models) or incidence rate ratio (negative binomial). Year-adjusted models additionally adjusted for procedure year as a continuous variable. CPB-only cohort (*n* = 329). Full attenuation of the furosemide effect with year adjustment indicates a strong parallel secular trend in diuretic use.

**Table 7:** STAT-Stratified analysis of ventilation days (fully adjusted).

STAT stratum	*n* Pre	*n* Post	Vent Pre, mean (median)	Vent Post, mean (median)	IRR (95% CI), *p*
STAT 1–2 (lower complexity)	222	56	7.42 (2.0)	2.33 (0.0)	0.64 (0.45–0.92), *p* = 0.017
STAT 3+ (higher complexity)	37	14	11.71 (5.0)	6.33 (3.0)	0.50 (0.24–1.04), *p* = 0.063

Negative binomial regression with adjustment for age, weight, and CPB time within each stratum. CPB-only cohort (*n* = 329). Interaction test (POST × STAT_HIGH) *p* = 0.97, indicating consistent protocol-associated reduction across complexity tiers.

**Table 8:** Postoperative laboratory values (days 1–3).

Laboratory Value	POD	Pre Mean ± SD	*n*	Post Mean ± SD	*n*	*p*
Sodium (mmol/L)	1	146.21 ± 4.02	320	144.47 ± 5.30	102	<0.001
	2	147.05 ± 5.94	329	142.86 ± 5.98	99	<0.001
	3	143.84 ± 5.70	323	141.09 ± 5.06	93	<0.001
Potassium (mmol/L)	1	3.70 ± 0.57	320	3.86 ± 0.60	102	0.007
	2	3.90 ± 0.44	329	4.04 ± 0.41	99	0.004
	3	3.72 ± 0.44	323	3.88 ± 0.42	93	0.002
Calcium (mg/dL)	1	9.94 ± 1.50	327	10.19 ± 1.61	102	0.139
	2	8.84 ± 1.13	329	9.02 ± 0.89	99	<0.001
	3	8.70 ± 0.81	323	9.10 ± 0.78	93	<0.001
Glucose (mg/dL)	1	150.00 ± 37.61	335	137.77 ± 28.41	110	0.003
	2	127.75 ± 33.08	336	115.70 ± 25.71	103	<0.001
Lactic acid (mmol/L)	1	2.53 ± 1.55	317	1.81 ± 1.02	106	<0.001
	2	1.83 ± 1.18	287	1.58 ± 0.75	93	0.211
BUN (mg/dL)	1	12.24 ± 5.78	319	11.22 ± 4.75	102	0.118

Values are mean ± SD. Complete-case analysis; ≤8.5% missing. POD 1 = day of first procedure.

**Table 8b:** Hypernatremia analyses: protocol effect and association With AKI (CPB cohort).

Analysis	Pre/Reference	Post/Comparison	Effect estimate	*p*
Any hypernatremia (Na > 145, POD 1–3)	200/254 (78.7%)	44/70 (62.9%)	*p* = 0.008	0.008
Persistent hypernatremia (≥2 of 3 days)	152/254 (59.8%)	27/70 (38.6%)	*p* = 0.002	0.002
Severe hypernatremia (Na > 148, any day)	144/254 (56.7%)	26/70 (37.1%)	*p* = 0.004	0.004
Post-protocol → hypernatremia (adj. age/wt/STAT/CPB)	Reference	Post-protocol	OR: 0.44 (0.22–0.89)	0.022
Hypernatremia → AKI (adj. age/wt/STAT/POST)	Normonatremic	Hypernatremic	OR: 1.36 (0.67–2.74)	0.39

Hypernatremia defined as serum Na >145 mmol/L. “Any hypernatremia” = any qualifying day during POD 1–3. “Persistent” = qualifying ≥2 of 3 days. “Severe” = Na >148 on any of POD 1–3. The hypernatremia-AKI association attenuates in the CPB cohort (OR: 1.36, NS) compared with the broader literature [Martinez-Garcia 2023; OR: 2.7 (21)], most likely because all patients in this cohort underwent CPB and therefore share a common upstream renal insult.

## Results

### Study population and baseline characteristics

After exclusion of 26 non-structural cases and 106 non-CPB lateral thoracotomy cases (coarctation repair and PDA ligation), 329 patients undergoing structural CHD surgery with cardiopulmonary bypass were included: 259 pre-protocol and 70 post-protocol (Table 9). Cohorts were well-balanced on age (4.30 ± 5.36 vs. 4.13 ± 5.17 years, *p* = 0.384) and weight (18.44 ± 20.73 vs. 20.09 ± 22.99 kg, *p* = 0.153). Mean cardiopulmonary bypass time was significantly shorter post-protocol (83.0 ± 60.2 vs. 101.2 ± 60.0 min, *p* = 0.008), while aortic cross-clamp time was not significantly different (47.7 ± 33.1 vs. 53.0 ± 40.6 min, *p* = 0.512). The STAT category distribution was not significantly different between groups (chi-square *p* = 0.248), with the majority of patients (84% pre, 80% post) classified as STAT 1 or 2. Modified ultrafiltration was not used in any patient in either cohort. Ventricular function on intraoperative transesophageal echocardiography (TEE) performed in the operating room after weaning from CPB (a purely intraoperative observation that precedes any postoperative fluid management) was depressed (mild, moderate, or severe) in 53/258 (20.5%) pre-protocol and 5/70 (7.1%) post-protocol patients (*p* = 0.008), reflecting the parallel reduction in CPB time and shifted case mix in the post-protocol era. All operative-period baseline differences were included as covariates in fully-adjusted sensitivity models (Tables 1, 2).

**Table 9:** Baseline patient characteristics (CPB cohort, *n* = 329).

Characteristic	Pre-Protocol (*n* = 259)	Post-Protocol (*n* = 70)	*p*-value
Age, years, mean ± SD	4.30 ± 5.36	4.13 ± 5.17	0.384
Weight, kg, mean ± SD	18.44 ± 20.73	20.09 ± 22.99	0.153
CPB time, min, mean ± SD	101.2 ± 60.0	83.0 ± 60.2	0.008
Cross-clamp time, min, mean ± SD	53.0 ± 40.6	47.7 ± 33.1	0.512
MUF use, *n* (%)	0 (0%)	0 (0%)	N/A
Post-CPB TEE: VF depressed, *n* (%)^1^	53/258 (20.5%)	5/70 (7.1%)	0.008
STAT 1, *n* (%)	62 (23.9%)	16 (22.9%)	0.248^2^
STAT 2, *n* (%)	160 (61.8%)	40 (57.1%)	
STAT 3, *n* (%)	27 (10.4%)	11 (15.7%)	
STAT 4, *n* (%)	10 (3.9%)	2 (2.9%)	
STAT 5, *n* (%)	0 (0%)	1 (1.4%)	

Values are mean ± SD or *n* (%). Mann–Whitney *U* (continuous); chi-squared or Fisher's exact (categorical).

1 Intraoperative transesophageal echocardiogram performed in the operating room after weaning from CPB (available for 258 pre-protocol and 70 post-protocol patients); a purely intraoperative observation included as an operative covariate.

2 *p*-value reflects overall STAT distribution comparison. STAT categories derived from primary diagnosis per Jacobs et al. (2009). Cohort restricted to patients undergoing cardiopulmonary bypass; lateral thoracotomy cases (coarctation, PDA ligation) excluded.

### Diuretic exposure

Total diuretic doses fell post-protocol, from 14.66 ± 8.32 (median 13) to 10.40 ± 6.81 (median 10; *p* < 0.001) (Table 5). Furosemide exposure declined from 9.72 ± 6.18 to 7.46 ± 5.28 mg/kg (23% reduction, median 8.8 vs. 7.1 mg/kg; *p* = 0.007). Unique diuretic agents per patient fell from 1.67 ± 0.87 to 1.29 ± 0.66 (*p* < 0.001). Chlorothiazide and spironolactone each declined significantly (*p* < 0.001 and *p* = 0.029, respectively). Bumetanide use was minimal in both cohorts (*p* = 0.615). The year-adjusted sensitivity analysis below identifies a parallel secular trend in diuretic use, and the protocol-attributable component of these reductions should be interpreted in that context.

### Clinical outcomes

**Figure 2:**
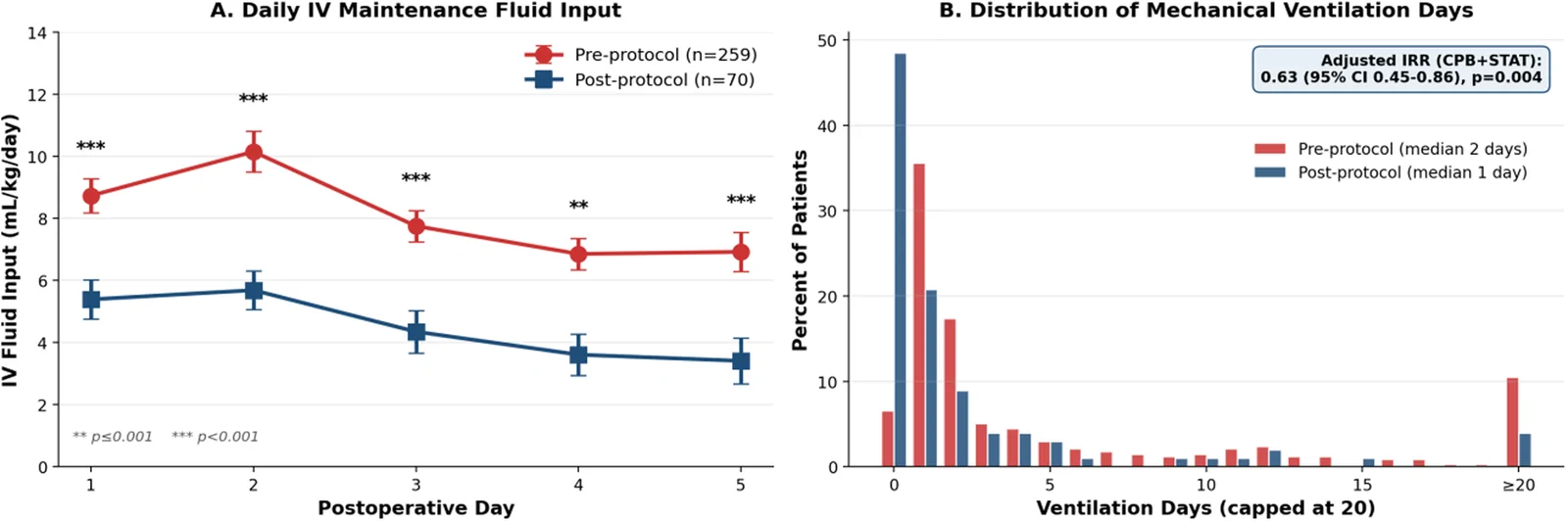
Fluid input trajectory and mechanical ventilation distribution. Panel **(A)** Mean daily IV maintenance fluid input (mL/kg/day) by postoperative day, pre- vs. post-protocol cohorts. Error bars show standard error of the mean. Post-protocol cohort received approximately 38% less per-kilogram IV maintenance fluid throughout the first five postoperative days, consistent with protocol implementation. Panel **(B)** Distribution of mechanical ventilation days by cohort, capped at 20 for display. Post-protocol cohort shows a substantial leftward shift, with 48% extubated by Day 0–1 vs. 6.5% pre-protocol. Adjusted incidence rate ratio (CPB time and STAT) shown in inset.

Mean hospital LOS decreased from 18.83 ± 24.93 to 12.33 ± 16.77 days (median 8 vs. 6; *p* = 0.054, borderline) (Table 5b). ICU LOS declined from 18.42 ± 24.59 to 12.27 ± 16.76 days (*p* = 0.090). Ventilation days fell from 7.12 ± 15.18 (median 2) to 4.01 ± 9.10 days (median 1; *p* < 0.001) (Figure 2B). AKI incidence was 42.5% (110/259) pre-protocol and 37.1% (26/70) post-protocol (Fisher exact *p* = 0.49, not statistically significant in the CPB-only cohort). In-hospital mortality was low in both cohorts; formal comparison was not performed given limited events. The magnitude of the ventilation reduction is substantial; the LOS reduction did not reach conventional significance, and the multivariable and year-adjusted analyses below are critical for interpretation, as is comparison with prior literature in the Discussion.

### Multivariable analysis

Post-protocol status was independently associated with reduced hospital LOS (*β* = −7.34 days, 95% CI: −13.13 to −1.54; *p* = 0.013) and a directionally consistent reduction in ventilation days (*β* = −3.48 days, 95% CI: −7.08 to 0.12; *p* = 0.058) after adjusting for age, weight, and STAT category in the CPB cohort (Table 5c). The AKI association did not reach statistical significance in the primary model (OR: 0.67, 95% CI: 0.36–1.22; *p* = 0.190). Age was a strong independent predictor of AKI (OR: 0.68 per year, *p* < 0.001) and STAT category was an independent predictor of both AKI (OR: 1.42 per category, *p* = 0.047) and LOS (*β* = +5.32 days per category, *p* = 0.002), confirming STAT's validity as the primary operative-complexity adjustment in this CPB cohort.

### Sensitivity analysis 1: year-adjusted models

In the year-adjusted sensitivity analysis (CPB cohort, *n* = 329), post-protocol status remained independently associated with reduced LOS (*β* = −19.57 days, 95% CI: −29.19 to −9.95; *p* < 0.001), and the AKI association reached statistical significance (OR: 0.26, 95% CI: 0.09–0.71; *p* = 0.009) (Table 5d). Procedure year emerged as a positive predictor of both LOS (*β* = +2.89/year, *p* = 0.002) and AKI (OR: 1.26/year, *p* = 0.022), reflecting that the year-adjusted protocol estimate isolates the protocol-attributable component from a counter-directional secular trend within this CPB-restricted cohort. A statistically significant year effect was identified for furosemide mg/kg (*β* = −0.64/year, *p* = 0.002), consistent with a parallel secular trend toward reduced diuretic use independent of the protocol; the protocol-attributable component of the diuretic reduction is therefore likely smaller than the unadjusted point estimate suggests.

### Sensitivity analysis 2: Log-transformed and generalized linear models

Because LOS and ventilation days are right-skewed, generalized linear models with log link were performed as a prespecified sensitivity analysis. For LOS and furosemide mg/kg, gamma regression with a log link was used; for ventilation days, negative binomial regression. All models adjusted for age, weight, and STAT category. Results are reported as ratios of geometric means (gamma models) or incidence rate ratios (negative binomial) with 95% confidence intervals (Table 6b). The post-protocol cohort showed a 34% lower geometric mean hospital LOS (ratio: 0.66, 95% CI: 0.50–0.88; *p* = 0.005), a 49% lower expected ventilation-day count (IRR: 0.51, 95% CI: 0.37–0.69; *p* < 0.001), and 28% lower geometric mean furosemide mg/kg exposure (ratio: 0.72, 95% CI: 0.64–0.83; *p* < 0.001). Effect direction and statistical significance were consistent with the primary linear regression results, supporting robustness of the findings to outcome distribution. Year-adjusted GLM models further reduced the LOS effect (ratio: 0.33, 95% CI: 0.21–0.53; *p* < 0.001) and the ventilation effect (IRR: 0.23, 95% CI: 0.14–0.39; *p* < 0.001), with year emerging as a positive predictor; the furosemide effect attenuated to non-significance (ratio: 0.93; *p* = 0.55), consistent with the secular diuretic trend identified in the linear year-adjusted model.

### Sensitivity analysis 3: fully adjusted models with CPB time and STAT category

As a more rigorous test of operative-complexity confounding, we performed a fully-adjusted sensitivity analysis incorporating CPB time as an additional operative covariate alongside age, weight, and STAT mortality category in the CPB-only cohort (*n* = 329, with CPB time available for 100% of analyzed patients). With this adjustment, several previously significant associations attenuate substantially (Table 1). The AKI association did not reach statistical significance in the fully-adjusted model (OR: 0.87, 95% CI: 0.46–1.63; *p* = 0.66), with CPB time emerging as a strong independent predictor (OR: 1.01 per min, 95% CI: 1.005–1.016; *p* < 0.001). The LOS association attenuated to borderline significance (ratio: 0.76, 95% CI: 0.58–1.00; *p* = 0.052). The ventilation-day association remained statistically significant (IRR: 0.63, 95% CI: 0.45–0.86; *p* = 0.004). The furosemide mg/kg association remained robust in the primary fully-adjusted model (ratio: 0.75, 95% CI: 0.65–0.85; *p* < 0.001) but was fully attenuated when procedure year was added (ratio: 0.97; *p* = 0.77), with year now the dominant predictor (ratio: 0.94 per year; *p* = 0.003). In year-adjusted fully-adjusted models, the LOS and AKI associations reached statistical significance, but with procedure year emerging as a positive predictor of outcomes (year ratio for LOS 1.18 per year, *p* < 0.001; year OR for AKI 1.31 per year, *p* = 0.010), reflecting that the year-adjusted protocol estimate isolates the protocol-attributable component from a partially counter-directional secular trend. Taken together, these findings indicate that a substantial portion of the apparent protocol effect on AKI and LOS, but not on ventilation days, is explained by shorter CPB times in the post-protocol era.

To summarize the robustness of the primary outcome to progressive adjustment, the ventilation-day effect across all model specifications is presented in Table 10. The association remained statistically significant at every level of adjustment, including the most stringent model incorporating CPB time, STAT category, and post-CPB intraoperative TEE ventricular function.

**Table 10:** Ventilation-Day association across progressive adjustment levels (CPB cohort).

Model (adjustment set)	IRR (95% CI)	*p*-value
Primary (age, weight, STAT)	0.51 (0.37–0.69)	<0.001
+ CPB time	0.63 (0.45–0.86)	0.004
+ CPB time + post-CPB TEE ventricular function	0.69 (0.50–0.96)	0.026
+ procedure year (secular-trend adjusted)	0.30 (0.18–0.50)	<0.001

Negative binomial regression in the CPB-only cohort (*n* = 329). IRR = incidence rate ratio for ventilation days (post-protocol vs. pre-protocol); values below 1.0 indicate fewer expected ventilation days post-protocol. The primary model is the prespecified primary analysis; the remaining rows are prespecified sensitivity analyses. The association remained statistically significant across all adjustment levels. The year-adjusted estimate is more extreme because procedure year acted as a counter-directional predictor within this cohort, as detailed in the year-adjusted analysis above.

### Subgroup analysis by surgical complexity

**Figure 3:**
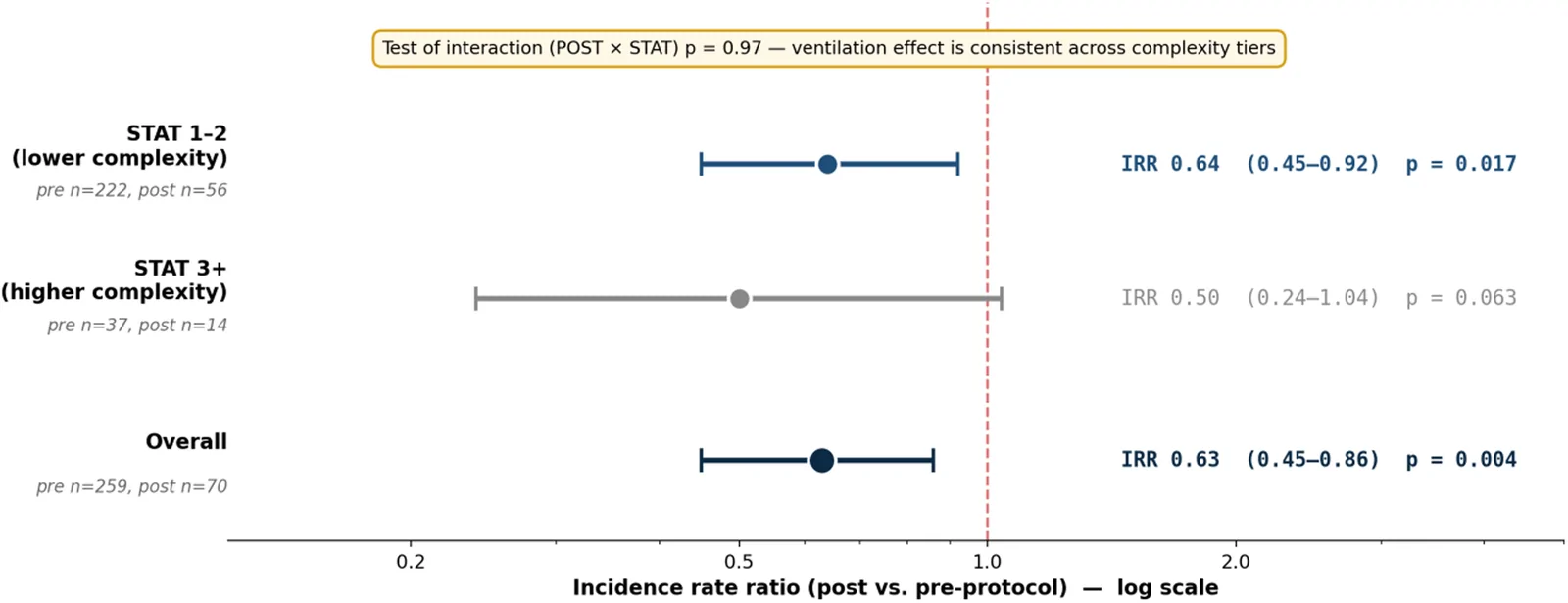
Ventilation-day effect stratified by STAT mortality category. Forest plot of the negative binomial incidence rate ratio for mechanical ventilation days (post vs. pre-protocol) stratified by STAT mortality category in the CPB-only cohort (*n* = 329). Estimates are from negative binomial regression adjusted for age, weight, and CPB time within each stratum. The overall (pooled) estimate is shown for reference. Formal test of interaction (POST × STAT category) *p* = 0.97, indicating consistency of the protocol-associated ventilation reduction across complexity tiers despite the wider confidence interval in the smaller higher-complexity stratum (*n* = 14 post-protocol).

Because STAT mortality category was used as the primary operative-complexity covariate in this CPB-restricted cohort, prespecified subgroup analyses by complexity tier are presented as STAT-stratified analyses (Table 7; Figure 3). Specifically, because mechanical ventilation duration was the most robust outcome under full multivariable adjustment, we performed a prespecified subgroup analysis stratified by STAT mortality category (STAT 1–2 vs. STAT 3+) using negative binomial regression adjusted for age, weight, and CPB time within each stratum. The ventilation-day association was statistically significant in the lower-complexity stratum (STAT: 1–2: IRR: 0.64, 95% CI: 0.45–0.92; *p* = 0.017) and directionally consistent but not reaching significance in the higher-complexity stratum (STAT 3+: IRR: 0.50, 95% CI: 0.24–1.04; *p* = 0.063), the latter likely reflecting power limitations from the small post-protocol sample (*n* = 14). The formal test for interaction between protocol status and STAT complexity was not significant (*p* = 0.97), which is best interpreted as an absence of evidence for effect modification rather than as positive evidence of a uniform effect, given the limited power of interaction testing in this sample.

### Fluid balance

Daily fluid input per kilogram was significantly lower post-protocol on every measured day through Day 5 (all *p* ≤ 0.001, except Day 4 *p* = 0.001) (Table 3; Figure 2A), consistent with implementation of the fluid restriction protocol. Post-protocol patients showed a trend toward higher urine output on Day 1 (28.81 vs. 23.45 mL/kg, *p* = 0.229) and significantly higher output on Day 2 (42.44 vs. 33.49 mL/kg, *p* = 0.047). The combination of lower input and equal-or-higher output is not attributable to increased diuretic use, given simultaneously lower diuretic exposure. While this pattern is biologically compatible with improved intrinsic renal perfusion under optimized intravascular volume conditions, intrarenal hemodynamics were not directly measured and this interpretation remains inferential.

### Laboratory values and electrolyte profile

Serum sodium was significantly lower post-protocol on Days 1–3 (all *p* < 0.001), consistent with reduced sodium loading from restricted IV fluids. Mean values remained within the laboratory reference range. The proportion of patients with mild hyponatremia (sodium <135 mmol/L) was numerically higher post-protocol but absolute rates remained low: 0.3% vs. 2.2% (POD 1; *p* = 0.14), 0.3% vs. 3.3% (POD 2; *p* = 0.04), and 1.3% vs. 4.7% (POD 3; *p* = 0.07). No patient in either cohort had severe hyponatremia (<130 mmol/L). Conversely, hypernatremia (>145 mmol/L) was substantially more common pre-protocol on POD 2 (57.7% vs. 31.9%) and POD 3 (30.5% vs. 20.0%), reflecting the historical sodium loading pattern. Potassium was significantly higher on Days 1–3 (all *p* < 0.01), reflecting reduced loop diuretic-driven kaliuresis. Calcium was higher on Days 2 and 3 (both *p* < 0.001). Glucose was lower on Days 1 and 2 (*p* = 0.003 and *p* < 0.001). Day 1 lactate was significantly lower post-protocol (1.81 ± 1.02 vs. 2.53 ± 1.55 mmol/L, *p* < 0.001) (Table 8). BUN did not differ significantly.

### Multiple imputation sensitivity analysis for laboratory outcomes

To assess whether the complete-case lab analyses were materially biased by missingness, a prespecified multiple imputation sensitivity analysis was performed using iterative chained-equations imputation (10 imputations conditioned on protocol status, age, weight, CPB time, STAT category, and the full lab covariate matrix). Estimates were pooled using Rubin's rules and are compared with complete-case estimates in Table 4b. Pooled imputed estimates were closely concordant with complete-case results for both mean sodium values and hypernatremia proportions on each postoperative day (within 0.1 mmol/L for means and within 1 percentage point for the hypernatremia threshold). The directional and quantitative consistency between approaches supports the robustness of the lab-based findings, including the protocol-associated reduction in postoperative hypernatremia, and indicates that missingness does not materially alter the interpretation of the electrolyte analyses.

### Post-CPB intraoperative TEE ventricular function: A Key operative covariate

Ventricular function on intraoperative transesophageal echocardiography performed in the operating room after weaning from cardiopulmonary bypass and before transfer to the cardiac ICU was available for 328 of 329 analyzed patients (99.7%; 258 pre-protocol, 70 post-protocol). Because this assessment is performed in the operating room before any postoperative fluid management has occurred, post-CPB TEE ventricular function is a purely intraoperative observation that captures the operative trajectory and serves as an additional operative covariate alongside CPB time and STAT category. The post-protocol cohort had a lower prevalence of post-CPB ventricular dysfunction (7.1% vs. 20.5%, *p* = 0.008), most plausibly reflecting the parallel reduction in CPB time and shifted case mix in the post-protocol era rather than any protocol effect, given that the assessment precedes protocol implementation in time.

Post-CPB TEE ventricular dysfunction carried substantial independent prognostic weight as an operative-state predictor of subsequent fluid management and outcomes. Patients identified at the moment of CPB separation as having depressed function received significantly more IV maintenance fluid on POD 1 (mean 13.9 vs. 8.0 mL/kg, *p* = 0.005) and POD 2 (17.8 vs. 8.4 mL/kg, *p* < 0.001), consistent with appropriate clinical practice of supporting cardiac output with additional preload when post-CPB systolic function is reduced. The intraoperative TEE finding therefore directly informs ICU fluid management decisions: clinicians, knowing that a patient came off bypass with depressed ventricular function, appropriately liberalize fluid administration regardless of protocol intent. After adjustment for age, weight, and STAT category, post-CPB VF depression was independently associated with AKI (OR: 3.01, 95% CI: 1.51–5.99; *p* = 0.002), longer ventilation (IRR: 3.32, 95% CI: 2.43–4.55; *p* < 0.001), and longer LOS (gamma ratio: 2.16, 95% CI: 1.58–2.94; *p* < 0.001). Unadjusted AKI in patients with post-CPB VF depression was 70.7% vs. 34.8% in patients with good post-CPB function (RR 2.03), reflecting the strong link between post-CPB cardiac state and renal injury via the low cardiac output pathway.

When post-CPB VF status is added as an operative covariate to the protocol-AKI model in the VF-available subset (*n* = 328), the protocol-attributable AKI association attenuates from OR: 0.67 to 0.80 (95% CI: 0.43 to 1.48; *p* = 0.48), and to OR: 0.91 (95% CI: 0.48–1.71; *p* = 0.77) when CPB time is additionally included. The protocol-ventilation association also attenuates but remains statistically significant: IRR: 0.68 (95% CI: 0.49–0.93; *p* = 0.015) with post-CPB VF in the model, and IRR: 0.69 (95% CI: 0.50–0.96; *p* = 0.026) with post-CPB VF and CPB time both included. The persistence of the ventilation-day effect across all adjustments, including this clinically critical operative-state confounder, supports the inference that fluid restriction independently shortens ventilatory support beyond what is explained by case mix, operative complexity, CPB duration, or post-CPB cardiac state. The attenuation of the AKI association under post-CPB VF adjustment is consistent with VF status being a strong operative-period predictor of postoperative renal injury, and its inclusion in the sensitivity model is appropriately conservative.

## Discussion

In this retrospective single-center before-and-after cohort of 329 pediatric patients undergoing structural CHD surgery with cardiopulmonary bypass, post-protocol status was associated with reduced diuretic exposure and shorter hospital and ventilation durations. These findings are observational and hypothesis-generating; the before-and-after design does not permit attribution of the observed associations to the fluid restriction protocol specifically, as discussed below. Multivariable models adjusting for age, weight, and STAT category yielded estimates that remained statistically significant for diuretic exposure, LOS, and ventilation days; the AKI association did not reach statistical significance either unadjusted (42.5% vs. 37.1%, *p* = 0.49) or in any adjusted model. Log-transformed sensitivity analyses for skewed outcomes were directionally consistent with the primary models. The interpretation that follows emphasizes the limits of causal inference inherent to the design and the magnitude of the observed estimates.

### Mechanistic considerations

Cardiopulmonary bypass exposes circulating blood to the artificial bypass circuit surface, initiating activation of complement, coagulation, and kallikrein cascades (2, 4). The resulting inflammatory mediators, particularly IL-6, IL-8, and TNF-α, disrupt tight junctions between endothelial cells and increase vascular permeability (3). Plasma proteins and fluid leak into the interstitial space. Crystalloid pump prime dilutes plasma oncotic pressure, amplifying the Starling forces driving further fluid extravasation (2). These mechanisms are well established and are presumed to underpin the rationale for fluid restriction strategies. The Starship-derived protocol restricts the exogenous fluid load that may amplify CPB-induced extravasation, rather than relying on diuresis to remove accumulated interstitial fluid (4, 15).

Intraoperative post-CPB transesophageal echocardiography emerged as a clinically important operative covariate in the protocol-outcome relationship. Patients identified at the moment of CPB separation as having depressed systolic function received more IV maintenance fluid in the early postoperative period (POD 1: 13.9 vs. 8.0 mL/kg, *p* = 0.005; POD 2: 17.8 vs. 8.4 mL/kg, *p* < 0.001), reflecting appropriate clinical practice of supporting cardiac output with additional preload when post-CPB function is reduced. Post-CPB VF depression was also itself a strong independent predictor of AKI (OR: 3.01), prolonged ventilation (IRR: 3.32), and longer LOS (ratio: 2.16), reflecting the well-established mechanism of low cardiac output driving renal hypoperfusion and pulmonary edema. After adjustment for post-CPB ventricular function, CPB time, and STAT category, the protocol-attributable AKI association no longer reaches statistical significance (OR: 0.91; *p* = 0.77), consistent with these operative-state covariates jointly capturing the major determinants of postoperative renal injury. The ventilation-day association, by contrast, persists under all adjustments including post-CPB VF status (IRR: 0.69, 95% CI: 0.50–0.96; *p* = 0.026), supporting the inference that fluid restriction independently shortens ventilatory support through a mechanism that is not fully explained by case mix, operative complexity, or post-CPB cardiac state.

### Fluid balance and inferred renal perfusion

Post-protocol patients received significantly less fluid per kilogram on Days 1–5, while showing a trend toward higher urine output on Day 1 and significantly higher output on Day 2, despite lower diuretic use. The directional pattern of reduced input with preserved or improved output, in the absence of increased pharmacologic support, is biologically compatible with improved glomerular filtration through avoidance of elevated central venous pressure. Fluid overload after CPB has been shown to raise right atrial pressure, reduce the renal perfusion pressure gradient, and impair filtration in the cardiac surgery-AKI literature (7, 8). The lower Day 1 lactate in post-protocol patients (1.81 vs. 2.53 mmol/L, *p* < 0.001) is consistent with improved early tissue oxygen delivery. These mechanistic interpretations remain inferential, as cardiac output, central venous pressure, and intrarenal hemodynamics were not directly measured in this retrospective cohort. The findings are hypothesis-consistent rather than mechanistically demonstrative.

### Magnitude of effect and plausibility

The magnitude of effect estimates, particularly the approximately 34% reduction in mean LOS and approximately 44% reduction in mean ventilation days in the unadjusted CPB cohort comparison, is substantial for a single perioperative intervention. Effect sizes of this approximate magnitude are uncommon in the perioperative fluid management literature: prior pediatric studies of fluid balance interventions and bundled care pathways have generally reported more modest reductions, on the order of 1–3 days for LOS and 1–2 days for ventilation (9–11, 14, 19). The dispersion of LOS in the pre-protocol cohort (SD 24.93) was driven in part by a long right tail of complicated cases, and reductions in mean LOS may overstate typical clinical impact relative to median changes (median: 8–6 days). The fully-adjusted ventilation-day effect (IRR: 0.63 with CPB time alone, IRR: 0.69 with additional adjustment for post-CPB intraoperative TEE ventricular function) represents the more conservative and methodologically robust estimate that is appropriate for clinical extrapolation.

The fully-adjusted sensitivity analysis incorporating CPB time and STAT category (Table 1c) substantially refines the interpretation of these effect magnitudes. CPB time emerged as a strong and consistent independent predictor of AKI (OR: 1.01 per min, *p* < 0.001), LOS (ratio: 1.006 per min, *p* < 0.001), and ventilation days (IRR: 1.009 per min, *p* < 0.001). Adjusting for CPB time and STAT, the protocol-attributable association with AKI was no longer statistically significant (OR: 0.87; *p* = 0.66), and the LOS association attenuated to borderline (ratio: 0.76; *p* = 0.052). The ventilation-day association remained statistically significant after full adjustment (IRR: 0.63; *p* = 0.004), and the furosemide mg/kg association remained robust in the primary fully-adjusted model. The fact that mean CPB time was nearly 20 min shorter post-protocol (83.0 vs. 101.2 min; *p* = 0.008) accounts for a meaningful portion of the apparent protocol effect on AKI and LOS. This finding does not diminish the clinical relevance of the observed outcome improvements, which are real and clinically important; it does, however, indicate that a substantial part of the improvement reflects evolving operative practice rather than fluid restriction alone.

One important caveat to this interpretation is that CPB time adjustment may control for too much of the protocol's causal pathway. If the fluid protocol acts on AKI partly through reduced postoperative sodium loading and hypernatremia avoidance, as discussed in detail below, then this mechanism is mediated through postoperative variables that occur downstream of CPB and are not captured by CPB time as a covariate. The statistical attenuation of the protocol-AKI association after CPB time adjustment is therefore consistent with two possibilities that are not mutually exclusive: (1) much of the apparent association reflects parallel shortening of CPB time, or (2) the protocol's effect operates through a postoperative electrolyte and fluid-balance mechanism that the fully-adjusted model does not fully attribute to the protocol. The absolute AKI rate of 37.1% post-protocol, which sits within the published range for pediatric cardiac surgery (see AKI Incidence in Context, below), and the well-established association between hypernatremia and AKI in this population (21–23), support the second possibility as a contributor.

### Temporal confounding and secular trends

The mean CPB time was significantly shorter post-protocol (83.0 vs. 101.2 min, *p* = 0.008), warranting specific consideration as a marker of evolving operative practice over the study period. The STAT case-mix distribution did not differ significantly between cohorts (chi-square *p* = 0.248), and modified ultrafiltration was not used in any patient in either cohort, eliminating MUF as a confounder. The implications of the shorter CPB time for interpreting the observed associations are addressed in the temporal confounding discussion below.

The year-adjusted sensitivity analysis showed that procedure year was not an independent predictor of LOS (*β* = +1.15/year, *p* = 0.180) or AKI (OR: 1.13/year, *p* = 0.157) in the primary STAT-adjusted models. However, in the fully-adjusted analysis incorporating CPB time, year emerged as a positive predictor of LOS (ratio: 1.18/year, *p* < 0.001) and AKI (OR: 1.31/year, *p* = 0.010), with shorter CPB times driving the apparent secular improvement. The post-protocol estimate in the fully-adjusted year-adjusted model isolates the protocol-attributable component beyond this trend. The secular decline in furosemide use across years (*β* = −0.41 mg/kg per year, *p* = 0.034; fully-adjusted year coefficient ratio: 0.94 per year, *p* = 0.003), independent of protocol status, indicates a parallel shift in diuretic stewardship at the institutional level, consistent with broader literature recommending more conservative diuretic use in postoperative cardiac patients (20). The full attenuation of the post-protocol furosemide coefficient in year-adjusted fully-adjusted models (ratio: 0.97; *p* = 0.77) suggests that the protocol-attributable component of the diuretic reduction is small relative to this broader secular trend.

Year adjustment captures linear secular trends but cannot fully account for non-linear or unmeasured changes in practice. A consideration warranting particular emphasis is that protocol implementation overlapped entirely with the later study period (post-protocol cases from May 2022 onward; pre-protocol cases April 2017 through April 2022), so the protocol period and the contemporary era of institutional practice are not statistically separable in this dataset. Several specific concurrent influences merit explicit acknowledgment as potential confounders of the ventilation and LOS associations. First, accumulating surgical and team experience over the eight-year period likely contributed to the observed reduction in mean CPB time (101.2–83.0 min) and to faster recovery. Second, evolving extubation strategy, including a shift toward earlier and protocolized extubation and increased use of early or on-table extubation in lower-complexity cases, would directly shorten ventilation duration independent of fluid management. Third, evolution in sedation and analgesia practice toward lighter, protocolized, and opioid-sparing regimens would facilitate earlier extubation and shorter ICU stay. Fourth, broader ICU pathway changes, including lung-protective ventilation, delirium prevention bundles, early mobilization, and standardized nursing-led postoperative care, plausibly contributed to the same outcomes. None of these practice elements was captured at the patient level in the retrospective record, and none can be excluded as a contributor. The observed associations should therefore be interpreted as the joint product of the fluid restriction protocol and the contemporaneous evolution of surgical, sedation, extubation, and ICU practice, with a prospective controlled design required to isolate the protocol-attributable component.

These limitations are inherent to the retrospective before-and-after design. Prospective studies with standardized CPB management protocols and systematic documentation of perfusion parameters would allow more precise isolation of the fluid restriction effect.

### Surgical complexity stratification

STAT-stratified analyses showed directionally consistent associations across complexity tiers, with the interaction test (*p* = 0.97) best interpreted as an absence of evidence for effect modification rather than positive evidence of uniformity, given limited power. The small post-protocol sample in higher-complexity strata (*n* = 14 in STAT 3+) constrains subgroup inference. STAT mortality category was derived from primary diagnosis (18) rather than from full procedure-level STAT scoring, which is a constraint on residual confounding control.

### Electrolyte and renal findings

Mean serum sodium values were lower post-protocol on Days 1–3, consistent with reduced sodium loading from restricted IV fluids, and remained within the laboratory reference range. Mild hyponatremia (sodium <135 mmol/L) was numerically more common post-protocol (POD 1: 0.3% vs. 2.2%, *p* = 0.14; POD 2: 0.3% vs. 3.3%, *p* = 0.04; POD 3: 1.3% vs. 4.7%, *p* = 0.07), although no severe hyponatremia (<130 mmol/L) occurred in either cohort; this warrants surveillance, particularly in younger infants and patients receiving hypotonic enteral feeds. Higher mean potassium values post-protocol are consistent with reduced loop diuretic-driven kaliuresis. Postoperative hypernatremia (sodium >145 mmol/L) was substantially less common post-protocol: any hypernatremia on POD 1–3 fell from 78.7% (200/254) to 62.9% (44/70) (*p* = 0.008), persistent hypernatremia (≥2 of 3 days) from 59.8% to 38.6% (*p* = 0.002), and severe hypernatremia (>148 mmol/L) from 56.7% to 37.1% (*p* = 0.004). After adjustment for age, weight, STAT category, and CPB time, post-protocol status was associated with lower odds of hypernatremia (OR: 0.44, 95% CI: 0.22–0.89; *p* = 0.022), one of the more robust associations in this analysis and not explained by operative-complexity changes (Table 8b).

Hypernatremia is clinically consequential in this population: it has been associated with AKI in critically ill children [OR: 2.7 (21)] and with increased hospital mortality after CHD surgery [OR: 3.08–4.35 at 24–72 h (23)], and was identified alongside prolonged CPB duration and reduced furosemide sensitivity as an independent predictor of post-cardiac-surgery AKI in the 2025 Copenhagen infant cohort (22). Within the present CPB-only cohort, however, hypernatremia was not independently associated with AKI after adjustment for age, weight, STAT, and protocol status (OR: 1.36, 95% CI: 0.67–2.74; *p* = 0.39), in contrast to the directionally consistent unadjusted association (49.3% vs. 19.2% in the original full cohort). This attenuation likely reflects the shared upstream renal insult of cardiopulmonary bypass across all analyzed patients, which compresses the variance attributable to other covariates and makes the published OR of 2.7 most applicable to general pediatric ICU populations rather than this homogeneous CPB-exposed cohort. The lower hypernatremia burden after protocol implementation is consistent with reduced sodium loading, but the present data do not establish that it produces a downstream renal-outcome benefit, and no AKI benefit was detected.

With respect to AKI itself, the association did not reach significance even before adjustment (42.5% pre vs. 37.1% post, *p* = 0.49), and both absolute rates sit within the published range for pediatric cardiac surgery, which typically falls between 15% and 64% depending on diagnostic criteria, age, and case complexity (22, 24–26). Studies using comparable KDIGO criteria have reported rates of 36% to 62% (22, 24, 25), and the Neonatal and Pediatric Heart and Renal Outcomes Network has documented rates exceeding 40% at some centers (19). The present cohort's rates are therefore unremarkable in context, and the data neither demonstrate nor exclude a clinically meaningful protocol effect on AKI.

### Future directions and sample size considerations

Power calculations based on the observed effect sizes (alpha=0.05, two-sided, 80% power) inform the design of future prospective studies. Using the fully-adjusted effect estimates from Table 1 as the target, a prospective trial with 1:1 allocation would require approximately 146 patients total to detect the observed ventilation-day effect (IRR: 0.63), 362 patients to detect the furosemide mg/kg effect (ratio: 0.75), 392 patients to detect the borderline LOS effect (ratio: 0.76), and approximately 6,770 patients to detect the attenuated AKI effect (OR: 0.87). With 3:1 unbalanced allocation that better matches real-world implementation, total sample sizes increase by approximately one-third (195, 482, 523, and 9,026 patients respectively). Using the larger original observed effect estimates (e.g., OR: 0.55 for AKI before full operative adjustment), approximately 382–507 patients would suffice for AKI detection.

These calculations indicate that the current CPB cohort (*n* = 329) was adequately powered for the LOS, ventilation-day, and furosemide outcomes when the observed primary-model effect sizes are taken at face value, and was adequately powered for AKI under the original observed unadjusted relative risk reduction. However, the cohort is substantially underpowered to confirm or refute the much smaller fully-adjusted AKI effect, which may reflect either a genuinely absent protocol effect once operative complexity is accounted for, or a small true effect that this study cannot reliably detect. A pragmatic next step is a prospective multicenter cluster-randomized trial of approximately 200–300 patients per arm, powered for ventilation-day duration and LOS as primary outcomes, with AKI as a secondary outcome. Such a trial would also permit prespecified collection of CPB time, ventricular function, MUF protocols, inotropic and ventilation weaning strategies, addressing the residual operative-covariate gaps inherent to the present retrospective design. A trial of the magnitude required to definitively resolve the fully-adjusted AKI question (approximately 7,000–9,000 patients) is unlikely to be practical in this population and probably not justified given the modest expected effect.

### The case for randomized trials

The persistence of the ventilation-day and primary furosemide associations under adjustment, together with the association between post-protocol status and lower postoperative hypernatremia, provides a signal of plausible clinical benefit sufficient to justify formal randomized evaluation. The findings reported here should be viewed as motivation for randomized trials rather than as a substitute for them. Three features of the present results are particularly relevant in this regard. First, the ventilation-day reduction is large enough in magnitude (point estimate IRR: 0.51 in the primary model; IRR: 0.69 after the most stringent adjustment for CPB time, STAT category, and post-CPB intraoperative TEE ventricular function) to be clinically meaningful if confirmed, with corresponding implications for sedation exposure, ventilator-associated complications, ICU length of stay, family experience, and direct hospitalization costs. Second, the intervention itself is low-cost, low-risk, scalable, and implementable without specialized equipment, making it a particularly attractive candidate for pragmatic cluster-randomized evaluation across diverse pediatric cardiac surgery centers. Third, several methodological gaps inherent to the present retrospective design, including incomplete operative-covariate capture for some perfusion variables, the inability to formally test bedside adherence, the inability to disentangle protocol effects from concurrent secular changes in surgical experience and ICU care, and the limited statistical power for AKI and high-complexity subgroups, are all directly addressable in a prospective randomized design with prespecified data collection. The pediatric cardiac surgery field has converged on the importance of reducing fluid overload after CPB through multiple lines of evidence [Hassinger 2014 (8), Alobaidi 2018 (9), Bellos 2020 (10), Hazle 2013 (11), Hanot 2019 (16), Kwiatkowski 2023 (27)], but no large randomized trial of postoperative fluid restriction has been published in this population. We hope this manuscript will encourage investigators and pediatric cardiac surgery networks (including the Pediatric Cardiac Critical Care Consortium, the Pediatric Acute Care Cardiology Collaborative, and the Neonatal and Pediatric Heart and Renal Outcomes Network) to consider a pragmatic cluster-randomized trial of protocolized postoperative fluid restriction as a logical and feasible next step. The infrastructure to conduct such a trial exists; the present observational signal, together with the corroborating literature, supports the rationale.

### Comparison With prior literature

The fully-adjusted ventilation-day effect (IRR: 0.63, approximately a 37% reduction in expected ventilation days; IRR: 0.69 with additional adjustment for post-CPB intraoperative TEE ventricular function, approximately 31% reduction) is directionally consistent with and quantitatively comparable to prior pediatric perioperative fluid management studies (Table 11). Hassinger et al. reported that early postoperative fluid overload preceded AKI and was associated with prolonged ventilation in pediatric cardiac surgery patients (8). The 2018 meta-analysis by Alobaidi et al. (33 studies, 12,902 children) found that each 1% increase in cumulative fluid balance was associated with a 6% increase in the odds of prolonged ventilation (9). The Bellos et al. 2020 systematic review (12 studies, 3,111 pediatric CHD patients) reported a dose-response relationship between fluid overload and adverse outcomes including prolonged ventilation (10). Kwiatkowski et al.'s randomized comparison of peritoneal dialysis vs. furosemide for fluid overload prevention in infants demonstrated that more aggressive fluid removal shortened ventilation by approximately 1 day (27). The present observational estimate is within the range of these prior reports, and unlike prior reports, was robust to adjustment for CPB time and post-CPB intraoperative TEE ventricular function, the dominant operative drivers of postoperative morbidity.

**Table 11:** Comparison of observed effects With prior pediatric fluid management studies.

Study	Design	Population	Reported effect on ventilation/LOS	Direction
Hassinger 2014 (8)	Retrospective cohort	*n* = 98 infants post CHD surgery	FO associated with prolonged ventilation and AKI	FO worse
Alobaidi 2018 (10) (meta-analysis)	33 studies, 12,902 children	Critically ill children	6% OR increase per 1% FO for prolonged ventilation	FO worse
Bellos 2020 (13) (meta-analysis)	12 studies, 3,111	Pediatric CHD postop	Dose-response of FO with adverse outcomes	FO worse
Kwiatkowski 2017 (20) (RCT)	Randomized trial	*n* = 73 infants post CHD	Peritoneal dialysis shortened ventilation by ∼1 day vs furosemide	Fluid removal beneficial
Present study (fully adjusted)	Retrospective cohort	*n* = 329 CPB cohort	IRR: 0.63 (95% CI: 0.45–0.86); ∼37% reduction in expected ventilation days	Fluid restriction beneficial

FO, fluid overload. The present study's effect estimate is directionally consistent with prior literature and remains significant after adjustment for CPB time, an operative covariate not adjusted for in most prior observational reports.

### Clinical implications

If the observed ventilation-day association reflects a protocol-attributable effect, the clinical implications would be meaningful. A reduction of this magnitude, applied to a population with median 2 ventilation days pre-protocol, would translate to roughly 1–2 fewer ventilator-days per patient, with corresponding reductions in sedation and analgesic exposure, ventilator-associated complication risk, unplanned extubation events, ICU bed-day occupancy, and family separation, and with earlier mobilization and enteral nutrition. The protocol carries minimal direct cost. These projections are contingent on the association being causal, which the present design cannot establish. Practical implementation guidance for centers adopting this approach, including order-set integration, sodium monitoring intervals, and enteral feeding advancement, is summarized in Box 1.

Box 1 **Protocol Implementation Tips for Clinicians**

Practical Pearls for Adopting Fluid Restriction After Pediatric Cardiac Surgery

- Start with 50% of Holliday-Segar maintenance on the day of bypass, advancing to 70% on POD 1 and titrating toward full maintenance as enteral feeds advance.
- Use isotonic crystalloid as the maintenance solution; avoid hypotonic fluids and D5W to limit free-water-related hyponatremia.
- Defer elective electrolyte supplementation for the first 24 h unless KDIGO or symptomatic thresholds are met.
- Avoid prophylactic diuretics; reserve furosemide for documented fluid overload >5% by daily weight or persistent oliguria after hemodynamic stabilization.
- Initiate trophic enteral feeds by POD 2 if hemodynamically stable; advance by 25% of goal per day and reduce IV maintenance proportionally.
- Monitor sodium every 4–8 h through POD 3; intervene early for either hypernatremia (Na >145) or hyponatremia (Na <135).
- Engage perfusion, anesthesia, surgical, ICU nursing, pharmacy, and dietetics teams before launch. Identify discipline-specific champions to sustain bundle fidelity.
- Embed the fluid restriction parameters directly into the postoperative cardiac surgery order set so that maintenance fluid prescribing is standardized by default rather than depending on individual clinician memory or audit-driven correction. Order-set integration substantially improves prescribing adherence, although bedside implementation should still be periodically audited. Track total IV intake including medication carriers, line flushes, and blood product volumes, not maintenance fluid alone. A complete order set, daily fluid calculator, and supporting audit tools are provided in Supplementary Implementation Toolkit.

From a renal safety perspective, the data do not suggest harm from fluid restriction, although they do not demonstrate an AKI benefit. The post-protocol AKI incidence of 37.1% sits within the published range for pediatric cardiac surgery [typically 15% to 64% (22, 24–26)] and was not statistically different from the 42.5% pre-protocol rate, either unadjusted (*p* = 0.49) or after adjustment. Concerns that aggressive fluid restriction might provoke pre-renal injury were not borne out in the observed data: AKI rates did not increase, urine output was not reduced, BUN did not differ, and Day 1 lactate was numerically lower post-protocol. Post-protocol status was associated with lower odds of postoperative hypernatremia after adjustment (OR: 0.44, 95% CI: 0.22–0.89; *p* = 0.022). Because hypernatremia has been associated with AKI and mortality in critically ill children and in pediatric cardiac surgery cohorts (21–23), a lower hypernatremia burden is plausibly favorable, but the present data cannot establish that the observed hypernatremia association translates into a renal-outcome benefit, and no such benefit was detected. Mild hyponatremia rates increased numerically (peak 4.7% on POD 3) and warrant monitoring, although no severe hyponatremia (<130 mmol/L) was observed.

### Economic implications

If even a fraction of the observed ventilation and LOS associations proves protocol-attributable in prospective evaluation, the economic implications would be favorable. Pediatric cardiac ICU bed-days are among the most expensive units of inpatient care, with reported costs of approximately 4,000–8,000 USD per ICU day and an additional ventilator surcharge of 1,500–3,000 USD per ventilator-day (28). Because the protocol carries minimal implementation cost (administrative, training, and order-set development), the cost-benefit ratio would likely be favorable even under conservative assumptions. A formal cost-effectiveness analysis is beyond the scope of this report and is a productive direction for future health-economics research.

### Equity and generalizability considerations

Several considerations bear on the equity and generalizability of these findings. The study population is drawn from a single mid-sized United States community-tertiary center serving a demographically diverse catchment area, including patients from urban, rural, and tribal communities in the surrounding region. The protocol's reliance on a structured calculator and a written order set may reduce variation in fluid management attributable to clinician experience, training, or unit-level practice culture, which is potentially equity-promoting. The protocol does not require any specialized equipment, advanced perfusion technology, or expensive pharmaceuticals, making it implementable in resource-constrained settings, including international centers. However, the absence of granular socioeconomic data in our dataset limits formal analysis of whether the protocol's effect varies by insurance status, primary language, parental education, or rurality. Future prospective evaluations should prespecify analysis of these dimensions. Generalizability to highly specialized referral centers performing predominantly STAT 4 and 5 procedures (e.g., Norwood and complex single-ventricle surgery) is also uncertain, as our STAT 3 + subgroup was small (*n* = 51 in the CPB cohort). The directionally consistent ventilation effect in STAT 3+ (IRR: 0.50, *p* = 0.063) is encouraging but underpowered.

### Strengths

This study has several methodological strengths that are uncommon in prior observational reports of pediatric perioperative fluid interventions. First, the analytic cohort was restricted *a priori* to patients undergoing cardiopulmonary bypass, with lateral thoracotomy cases (coarctation repair and PDA ligation) excluded because their physiology and postoperative fluid management requirements differ fundamentally from those of CPB cases. This restriction produces a mechanistically coherent cohort in which CPB-induced capillary leak is the dominant driver of postoperative fluid retention. Second, CPB time and aortic cross-clamp time were retrieved from operative records for 100% of analyzed patients, an adjustment not performed in prior pediatric fluid restriction reports and one that is uniquely possible because of the *a priori* CPB-only inclusion criterion. Third, STAT mortality category was derived for all patients from primary diagnosis using the validated Jacobs classification (18). Fourth, the analysis incorporated four complementary modeling approaches: primary linear regression for transparency, log-transformed generalized linear models (gamma and negative binomial) appropriate for the right-skewed outcome distributions, year-adjusted models to address secular trends, and fully-adjusted models incorporating operative variables, with results presented across all four for full transparency (Figure 1). Fifth, modified ultrafiltration use was characterized and confirmed not to have occurred in any patient, eliminating MUF as a potential confounder. Sixth, prespecified subgroup analyses by STAT complexity tier with a formal interaction test (*p* = 0.97) demonstrated consistency of the ventilation-day association across the surgical complexity spectrum. Seventh, complete-case missingness for laboratory variables was below 8.5% with missingness independent of treatment group, and a prespecified multiple imputation sensitivity analysis (Table 4) confirmed concordance of pooled imputed estimates with complete-case results, supporting the robustness of the lab analyses to missing-data assumptions. Eighth, embedding the fluid restriction parameters into the mandatory institutional electronic order set substantially standardized maintenance fluid prescribing in the post-protocol cohort, an implementation strategy that contrasts favorably with most prior reports relying on voluntary clinician adherence; we acknowledge that order-set-anchored prescribing improves but does not absolutely guarantee bedside implementation in a retrospective dataset (see Limitations). Finally, standardized effect sizes (Cohen's *d* with 95% CI, Hedges’ *g*, Cliff's delta, and risk differences) are reported for all primary clinical outcomes (Tables 5, 5b) alongside *p*-values, facilitating cross-study comparison and characterization of effect magnitudes independent of statistical significance.

### Limitations

This study has several important limitations. First, the design is retrospective and non-randomized, with group assignment determined by calendar period; causal inference is therefore not supported. Second, residual temporal confounding cannot be excluded. Protocol implementation overlapped entirely with the later study period, so the protocol-attributable effect cannot be statistically separated from concurrent institutional practice evolution beyond what linear year adjustment captures. Specific concurrent influences include accumulating surgical experience (reflected in the shorter mean CPB time), evolving extubation strategy toward earlier and protocolized extubation, lighter and more protocolized sedation, and broader ICU pathway changes (lung-protective ventilation, delirium prevention, early mobilization, standardized nursing care). The observed associations should be understood as the joint product of the protocol and these contemporaneous practice changes. Third, although CPB time and cross-clamp time were available for all analyzed patients, other operative variables were not captured (prime volume, perfusion temperature, perioperative corticosteroids, inotrope strategies, ventilation weaning protocols), and STAT category derived from primary diagnosis does not capture all procedure-level complexity.

Fourth, AKI classification was based on KDIGO serum creatinine criteria only. Urine output criteria could not be applied because only daily total urine output was recorded (not the hourly values KDIGO requires), daily totals were missing for 37%–71% of patients depending on day, and missingness was strongly correlated with age (mean 1.22 years among patients with output documented vs. 9.69 years among those without), reflecting routine catheterization and hourly charting in infants but not older children. A 24-h-average rate is also uninterpretable here because the protocol itself reduces urine output by reducing input, independent of renal function. Creatinine-only criteria underdetect AKI relative to combined criteria, biasing observed associations toward the null; this direction is conservative with respect to the AKI conclusion.

Fifth, the post-protocol sample (*n* = 70) limits power for binary outcomes and subgroups even after adjustment. The non-significant adjusted AKI association is consistent with a true null, but a clinically meaningful effect that this study was underpowered to detect cannot be excluded; the same applies to the STAT 3 + subgroup (*n* = 14 post-protocol). Sixth, modest baseline differences in CPB time, ventricular function, and STAT distribution were adjusted for but residual confounding remains possible. Seventh, the secular diuretic stewardship trend indicates that the furosemide reduction is partly or largely attributable to practice change independent of the protocol. Eighth, mechanistic interpretations regarding cardiac output and renal perfusion are inferential, as direct hemodynamic measurements were unavailable. Finally, nutritional and growth outcomes during fluid restriction were not captured (29, 30) and warrant future assessment.

## Conclusion

Practical implementation guidance is provided in Box 1.

In this retrospective single-center cohort of structural pediatric CHD surgery patients, post-protocol status was associated with shorter mechanical ventilation duration (IRR: 0.63, 95% CI: 0.45–0.86; *p* = 0.004) and lower furosemide exposure (ratio: 0.75, 95% CI: 0.65–0.85; *p* < 0.001) after adjustment for cardiopulmonary bypass time and STAT mortality category. The ventilation-day association was the most robust finding and is compatible with the mechanistic rationale for proactive fluid restriction in CPB-exposed patients, in which avoidance of postoperative interstitial pulmonary edema may facilitate earlier extubation, although the observational design cannot confirm this mechanism. Post-protocol status was also associated with lower sodium loading, a shift away from hypernatremia, and fewer ICU days.

Apparent reductions in AKI incidence and hospital LOS attenuated substantially under full adjustment for CPB time and STAT category, with CPB time emerging as a strong independent predictor of both outcomes. This indicates that a meaningful portion of the unadjusted improvements in AKI and LOS reflects concurrent improvements in surgical efficiency rather than the fluid protocol *per se*. A parallel institutional secular trend toward more conservative diuretic stewardship was also identified and likely contributes to the observed furosemide reduction. These attenuations are scientifically informative: they isolate the operative-complexity contribution to postoperative outcomes and clarify which signals are independently attributable to fluid management.

These findings should be interpreted as associative and hypothesis-generating, not causal. The retrospective before-and-after design precludes definitive attribution, and protocol implementation overlapped entirely with the later study period, meaning that the protocol-attributable effect cannot be statistically separated from concurrent institutional practice evolution beyond what year adjustment captures. Accumulating surgical experience and concurrent advances in ICU care over the eight-year study period likely contributed to the observed outcomes alongside the fluid restriction protocol itself. Restriction to CPB cases with 100% complete operative covariate data, combined with order-set-anchored maintenance fluid prescribing in the post-protocol cohort, strengthens internal validity by reducing case-mix dilution and standardizing prescribed fluid management. Bedside implementation variability cannot be fully excluded in a retrospective dataset.

Nonetheless, the persistence of the ventilation-day and primary furosemide associations under adjustment, together with the association between post-protocol status and lower hypernatremia, provides a signal of plausible clinical benefit sufficient to motivate prospective randomized evaluation. The findings reported here should be viewed as motivation for randomized trials of postoperative fluid restriction in pediatric cardiac surgery rather than as a substitute for them. A pragmatic multicenter cluster-randomized trial of approximately 200–300 patients per arm, powered for ventilation duration and length of stay as primary outcomes with AKI and hypernatremia as secondary outcomes, and with prespecified collection of operative variables and long-term renal follow-up, would directly address the residual uncertainties identified here. The intervention is low-cost, low-risk, scalable, and implementable without specialized equipment, making it a particularly attractive candidate for pragmatic randomized evaluation. We hope this study will encourage investigators and pediatric cardiac surgery networks to undertake such a trial as a logical and feasible next step toward establishing protocolized postoperative fluid restriction as evidence-based practice.

## Data Availability

The raw data supporting the conclusions of this article will be made available by the authors, without undue reservation.
