# Supplementary material for: Fluid restriction after pediatric cardiac surgery is independently associated With shorter mechanical ventilation: a retrospective cohort study With adjustment for cardiopulmonary bypass time
Source: Front Pediatr. 2026 Aug 11;14:1860058. doi: 10.3389/fped.2026.1860058 (PMC13504627; doi:10.3389/fped.2026.1860058)
Supplement: Supplementary file 1 [file Supplementaryfile1.docx]

**Supplementary Implementation Toolkit**

*Fluid Restriction After Pediatric Cardiac Surgery: Adapted Starship Protocol*

This supplement provides a structured implementation toolkit derived from the protocol used in the present study. It is intended for institutions considering adoption of a Starship-derived fluid restriction protocol after pediatric cardiac surgery. All materials are adapted from Finucane and Hamer (2016) [3] with institutional refinements, and may be modified to suit local practice. Use of these materials does not require permission from the present authors; appropriate citation is appreciated.

# **1. Protocol Summary**

## **1.1 Indications**

- Pediatric patients (<18 years) admitted to the PICU after structural CHD repair under cardiopulmonary bypass.
- Apply from the day of bypass through postoperative Day 5 unless clinical instability or specific contraindications develop.
- For non-bypass cardiac surgery, use the 70% maintenance restriction (less aggressive variant).

## **1.2 Contraindications and Cautions**

- Hemodynamic instability requiring active fluid resuscitation: suspend the protocol and resume after stabilization.
- Active hemorrhage or massive transfusion: protocol does not apply; treat per local bleeding management guidelines.
- Documented baseline AKI (KDIGO Stage 2 or higher) preoperatively: individualize fluid management with nephrology consultation.
- Single-ventricle palliation with passive pulmonary blood flow (e.g., Glenn, Fontan): consider higher target preload; consult cardiology.
- Severe sepsis or septic shock developing postoperatively: revert to standard sepsis fluid resuscitation.

## **1.3 Core Protocol Elements**

The protocol consists of five evidence-supported components, applied as a bundle:

**Table S1. Protocol Components**

| **#** | **Element** | **Specification** |
| --- | --- | --- |
| 1 | Maintenance fluid restriction | Day of bypass: 50% of standard Holliday-Segar maintenance. POD 1–2: 50–70%. POD 3 onward: titrate toward full maintenance as enteral feeds advance. |
| 2 | Avoid hypotonic solutions | Use isotonic crystalloid (e.g., 0.9% saline or balanced crystalloid). Avoid D5W and hypotonic saline as primary maintenance. |
| 3 | Delayed electrolyte supplementation | Hold elective potassium, calcium, magnesium replacement during the first 24 postoperative hours unless clinically symptomatic or KDIGO criteria for severe deficiency are met. |
| 4 | Diuretics for specific indications only | No prophylactic diuretic use. Indications: documented fluid overload >5% by daily weight, oliguria persisting beyond 6 hours after stabilization, or echocardiographic evidence of biventricular volume loading. |
| 5 | Early enteral nutrition | Initiate trophic feeds on POD 2 if hemodynamically stable and bowel function returning. Advance to goal as tolerated; reduce IV maintenance proportionally. |

## **1.4 Monitoring Schedule**

- Hourly: heart rate, blood pressure, MAP, central venous pressure (if line in place), urine output, fluid input totals.
- Every 4 hours during POD 1: serum sodium, potassium, ionized calcium, lactate, glucose, hematocrit.
- Every 8 hours during POD 2–3: as above plus BUN and creatinine.
- Daily: weight (same scale, same time), strict fluid balance (input/output by source), abdominal exam, neurologic check.
- Continuous: pulse oximetry, ECG, end-tidal CO₂ if ventilated.

# **2. Daily Fluid Calculator**

Use the Holliday-Segar formula for standard maintenance, then apply the restriction factor for the relevant postoperative day.

## **2.1 Holliday-Segar Standard Maintenance (24 h)**

**Table S2. Standard Holliday-Segar Maintenance Rates**

| **Weight (kg)** | **Daily volume (mL/day)** | **Hourly rate (mL/h)** |
| --- | --- | --- |
| First 10 kg | 100 mL/kg/day | 4 mL/kg/h |
| Next 10 kg (10–20 kg) | Add 50 mL/kg/day | Add 2 mL/kg/h |
| Above 20 kg | Add 20 mL/kg/day | Add 1 mL/kg/h |

## **2.2 Restriction Factor by Postoperative Day**

**Table S3. Restriction Factor Schedule**

| **Postoperative Day** | **Restriction factor** | **Target IV maintenance** |
| --- | --- | --- |
| Day of bypass | 0.50 | 50% of HS maintenance |
| POD 1 | 0.50–0.70 | 50–70% of HS maintenance |
| POD 2 | 0.60–0.80 | 60–80% of HS maintenance |
| POD 3 | 0.70–1.00 (advance with EN) | Titrate up as enteral feeds advance |
| POD 4–5 | Full maintenance minus EN | Replace IV with enteral as tolerated |

## **2.3 Worked Examples**

Example A: 12 kg toddler on day of bypass.

- Standard HS maintenance = (10×100) + (2×50) = 1,100 mL/day = ~46 mL/h.
- Day-of-bypass restriction (50%) = 550 mL/day = ~23 mL/h.

Example B: 25 kg child on POD 1.

- Standard HS maintenance = (10×100) + (10×50) + (5×20) = 1,600 mL/day = ~67 mL/h.
- POD 1 at 60% = 960 mL/day = ~40 mL/h.

Example C: 6 kg infant on POD 2.

- Standard HS maintenance = 6×100 = 600 mL/day = ~25 mL/h.
- POD 2 at 70% = 420 mL/day = ~17.5 mL/h.

# **3. Sample Order Set**

The following can be adapted to the institutional electronic order entry system. Bracketed items require physician-specific entry.

## **3.1 Admission Orders (Day of Bypass)**

- Patient weight: [enter kg]
- Calculated Holliday-Segar maintenance: [enter mL/h]
- IV maintenance: 0.9% normal saline or balanced crystalloid at [50% of HS rate] mL/h, route IV continuous.
- Strict input and output every 1 hour.
- Daily weight at 0600, same scale.
- Hold elective potassium, calcium, magnesium supplementation until POD 1 morning labs.
- No prophylactic diuretics.

## **3.2 POD 1 Orders**

- Reassess fluid balance and weight change.
- If weight gain >3% of baseline OR clinical fluid overload (rales, hepatomegaly, peripheral edema): notify physician for diuretic consideration.
- IV maintenance: [50–70%] of HS rate based on clinical assessment.
- Initiate trophic enteral feeds at 5–10 mL/kg/day if hemodynamically stable, no abdominal concern, lactate <2.5 mmol/L.
- Labs: Na, K, ionized Ca, Mg, BUN, Cr, lactate, glucose, hematocrit every 8 hours.

## **3.3 POD 2–5 Orders**

- Advance enteral feeds by 25% of goal per day as tolerated.
- Reduce IV maintenance proportionally to enteral advancement.
- Daily labs: Na, K, ionized Ca, BUN, Cr.
- Reassess need for ongoing IV access by POD 3.
- Consider extubation readiness daily once weaning criteria met.

# **4. Monitoring & Safety Checklist**

Daily checklist to be completed by the primary nurse and reviewed by the attending intensivist. The protocol should be paused or modified if any “yes” safety trigger is identified.

**Table S4. Daily Safety Checklist**

| **Safety check** | **Yes (trigger)** | **No (continue)** |
| --- | --- | --- |
| Hemodynamic instability requiring active fluid resuscitation? | Pause protocol | Continue |
| Serum sodium <130 mmol/L? | Notify MD; consider isotonic correction | Continue |
| Serum sodium >150 mmol/L? | Notify MD; assess for inadequate hypotonic intake balance | Continue |
| Urine output <1 mL/kg/h for >6 h after stabilization? | Notify MD; reassess preload, consider diuretic | Continue |
| Weight gain >5% of baseline by POD 2? | Notify MD; consider diuretic | Continue |
| Serum creatinine rise meeting KDIGO Stage 2? | Nephrology consult; individualize | Continue |
| New lactate >4 mmol/L? | Notify MD; assess perfusion | Continue |
| Active bleeding or unplanned reintervention? | Suspend protocol | Continue |

# **5. Order-Set Integration and Optional Adherence Audit**

The strongest and most sustainable approach to protocol adherence is to embed the fluid restriction parameters directly into the institutional postoperative cardiac surgery electronic order set, so that compliant prescribing is the default rather than a behavior requiring ongoing audit. The institution from which the present cohort was drawn achieved 100% protocol adherence through this approach. Order-set integration eliminates voluntary clinician variation, reduces cognitive load for the prescribing team, and produces a permanent and trustworthy record of intent that can be analyzed retrospectively without separate adherence tracking. The supplementary audit metrics below are offered for institutions that cannot yet integrate the protocol into a mandatory order set, or that wish to retrospectively verify adherence during a transition period.

## **5.1 Recommended First Step: Order-Set Integration**

- Build a dedicated pediatric postoperative cardiac surgery order set in the EHR with the day-of-bypass restriction factor pre-applied and an embedded Holliday-Segar maintenance calculator.
- Set the order set as mandatory for all postoperative admissions from the cardiac OR. Use forcing functions to require selection of a specified-volume IV maintenance order from the restriction-aligned list rather than free-text entry.
- Build a Best Practice Advisory triggered by sodium <130 or >150 mmol/L.
- Require clinical-indication free-text entry for any diuretic order in the first 24 postoperative hours.
- With these built into the order set, formal patient-level adherence audits become largely unnecessary, although periodic spot-checks remain valuable for quality assurance.

## **5.2 Optional Patient-Level Adherence Metrics (For Non-Order-Set Implementations)**

- Day-of-bypass IV maintenance: target ≤50% of calculated HS maintenance. Adherent if actual mL/kg ≤0.50 × calculated mL/kg.
- POD 1 IV maintenance: target ≤70% of calculated HS maintenance.
- Hypotonic fluid administered as maintenance: target zero. Adherent if no D5W or hypotonic saline received as continuous maintenance.
- Prophylactic diuretic administered: target zero in first 24 h unless specific indication documented. Adherent if no diuretic given without documented indication.
- Trophic enteral feeds initiated by POD 2: target yes if no contraindication.

## **5.3 Adherence Categories**

- Full adherence: all 5 metrics met.
- Partial adherence: 3 or 4 of 5 metrics met.
- Non-adherence: ≤2 metrics met.

## **5.4 Override Documentation**

When clinical circumstances require deviation from the protocol (active resuscitation, hemorrhage, renal replacement therapy, or specific contraindications), document in the medical record: (1) the specific deviation, (2) the clinical reason, (3) the timestamp, (4) the responsible clinician, and (5) the planned duration before reassessing protocol resumption. Under order-set-enforced implementation, deviations are recorded in the standard medical record rather than as separate protocol-override events.

# **6. Implementation Tips and Common Pitfalls**

## **6.1 Multidisciplinary Engagement**

- Engage perfusion, anesthesia, surgical, ICU nursing, pharmacy, and dietetics teams before launch. Each role contributes to bundle fidelity.
- Identify clinical champions in each discipline. Sustainability requires distributed ownership.
- Hold a brief launch huddle (15–20 minutes) covering rationale, evidence, calculator use, and audit plan.

## **6.2 Order Set and EHR Integration**

- Build a dedicated pediatric postoperative cardiac surgery order set in the EHR with the restriction factor pre-applied and an embedded HS calculator.
- Use forcing functions where appropriate (e.g., require a clinical indication free-text field for any postoperative diuretic order in the first 24 hours).
- Build a Best Practice Advisory (BPA) for sodium <130 or >150 mmol/L flagged automatically.

## **6.3 Common Pitfalls**

- Drift in non-maintenance fluid volume: medication carriers, line flushes, arterial line keep-open fluids, and blood product volumes can substantially exceed maintenance. Track total IV intake, not maintenance alone.
- Anchoring on weight at admission: postoperative interstitial third-spacing can mislead weight-based fluid calculations on POD 1–2. Use preoperative dry weight rather than current weight when possible.
- Over-aggressive enteral advancement: feeds advanced too quickly can produce ileus and reverse the gains from earlier enteral exposure. Advance 25%/day of goal.
- Failure to monitor sodium trend: occasional measurements miss developing hyponatremia or hypernatremia. Maintain the q4-8h schedule through POD 3.

## **6.4 Patient and Family Communication**

- Explain the protocol to parents on POD 0 or 1: “We limit IV fluids to help your child avoid extra fluid buildup that can prolong ventilation and ICU stay. It is safe and the team will monitor labs frequently.”
- Address common concerns: dry mouth (oral care), perception of restriction (reassure that fluids are calculated to meet needs).
- Involve parents in noting clinical signs (extubation readiness, urine output) once families are reoriented to the ICU environment.

# **7. Suggested Quality Metrics**

Institutions implementing the protocol may wish to track the following outcome and process metrics for internal benchmarking and continuous quality improvement:

**Table S5. Quality Metrics Dashboard**

| **Metric** | **Reporting frequency** | **Target / benchmark** |
| --- | --- | --- |
| Ventilation days (median) | Monthly | Reduction vs. baseline |
| Hospital LOS (median) | Monthly | Reduction vs. baseline |
| AKI incidence (KDIGO) | Monthly | <30%; below institutional baseline |
| Hypernatremia (Na >145) prevalence POD 1–3 | Monthly | <50%; reduction vs. baseline |
| Severe hyponatremia (Na <130) | Monthly | 0% |
| Furosemide mg/kg per patient (median) | Quarterly | Reduction vs. baseline |
| Order-set utilization rate (post-CHD admissions) | Quarterly | 100% (target via mandatory order set) |
| Clinical deviations recorded for documented indications | Quarterly | Track trend; identify systemic drivers |
| 30-day readmission rate | Quarterly | No increase vs. baseline |

# **8. References**

1. Finucane K, Hamer M. Fluid, diuretic and nutrition management in children having cardiac surgery. Starship Clinical Guidelines. 2016. https://starship.org.nz.

2. Present manuscript (this study). Full citation to be added on acceptance.
