# Supplementary material for: Fluid restriction after pediatric cardiac surgery is independently associated With shorter mechanical ventilation: a retrospective cohort study With adjustment for cardiopulmonary bypass time
Source: Front Pediatr. 2026 Aug 11;14:1860058. doi: 10.3389/fped.2026.1860058 (PMC13504627; doi:10.3389/fped.2026.1860058)
Supplement: Supplementary file 2 [file Supplementaryfile2.docx]

**STROBE Statement Checklist**

*Items that should be addressed in reports of observational cohort studies*

This manuscript adheres to the STROBE (Strengthening the Reporting of Observational Studies in Epidemiology) guidelines for cohort studies. Page numbers refer to the main manuscript.

| **Section / Topic** | **Item #** | **Recommendation** | **Location in manuscript** |
| --- | --- | --- | --- |
| Title and abstract | 1(a) | Indicate the study design with a commonly used term in the title or abstract. | Title: "Retrospective Cohort Study." Abstract first paragraph. |
|  | 1(b) | Provide an informative and balanced summary in the abstract. | Structured abstract with Background, Objective, Methods, Results, Conclusions. |
| **Introduction** |  |  |  |
| Background/rationale | 2 | Explain the scientific background and rationale for the investigation. | Introduction, paragraphs 1–4. |
| Objectives | 3 | State specific objectives and any prespecified hypotheses. | Introduction, final paragraph; Methods, Statistical Analysis. |
| **Methods** |  |  |  |
| Study design | 4 | Present key elements of study design early in the paper. | Methods, Study Design and Population. Retrospective single-center cohort, before-and-after design. |
| Setting | 5 | Describe setting, locations, and relevant dates. | Methods. Presbyterian Hospital PICU, Albuquerque NM, USA. April 2017 to February 2025. |
| Participants | 6(a) | Cohort: eligibility criteria, sources, and methods of selection. | Methods. All pediatric patients <18y undergoing open surgical structural CHD repair. 26 non-structural cases excluded. |
|  | 6(b) | Matched studies: matching criteria and number of exposed/unexposed. | Not applicable; no matching. Calendar-based group assignment described. |
| Variables | 7 | Define all outcomes, exposures, predictors, confounders, effect modifiers. | Methods, Outcomes and Data Collection. Primary: ventilation days, furosemide mg/kg. Secondary: AKI, LOS, ICU LOS, fluid balance, electrolytes. |
| Data sources/measurement | 8 | For each variable, give sources of data and details of methods of assessment. | Methods. EHR retrieval. AKI by KDIGO serum creatinine criteria. CPB time and STAT from operative records. STAT derived from primary ICD-10 per Jacobs 2009. |
| Bias | 9 | Describe efforts to address potential sources of bias. | Methods, Statistical Analysis. Multivariable adjustment, year-adjusted sensitivity analysis, full operative adjustment, STAT subgroup analysis. |
| Study size | 10 | Explain how the study size was arrived at. | Methods. All eligible patients enrolled. Post hoc power analysis presented in Discussion (Future Directions). |
| Quantitative variables | 11 | Explain how quantitative variables were handled in the analyses. | Methods, Statistical Analysis. Skewed outcomes: linear regression and GLM with log link (gamma, negative binomial). Continuous covariates adjusted as continuous. |
| Statistical methods | 12(a) | Describe all statistical methods, including those used to control for confounding. | Methods, Statistical Analysis. Logistic, linear, and GLM with log link. Adjusted for age, weight, RACHS-1, procedure duration; sensitivity for CPB time, STAT, procedure year. |
|  | 12(b) | Describe any methods used to examine subgroups and interactions. | STAT-stratified analysis (Table 6b, Figure 3) with formal interaction test. |
|  | 12(c) | Explain how missing data were addressed. | Methods. Complete-case analysis with <8.5% missing for laboratory variables; missingness independent of treatment group. |
|  | 12(d) | If applicable, explain how loss to follow-up was addressed. | Not applicable; in-hospital outcomes only. |
|  | 12(e) | Describe any sensitivity analyses. | Methods. Year-adjusted, log-transformed/GLM, fully-adjusted with CPB and STAT, STAT-stratified. |
| **Results** |  |  |  |
| Participants | 13(a) | Numbers at each stage of the study. | Results. 461 raw → 26 non-structural excluded → 435 structural → 106 non-CPB lateral thoracotomy excluded a priori → 329 CPB cases analyzed (259 pre, 70 post). CPB time and CCT available for 100% of analyzed patients. |
|  | 13(b) | Reasons for non-participation at each stage. | Methods. 26 non-structural cases excluded with diagnostic codes listed. |
|  | 13(c) | Flow diagram if applicable. | Cohort selection described in text; no separate flow diagram. |
| Descriptive data | 14(a) | Characteristics of study participants and information on exposures and potential confounders. | Table 1. Including age, weight, sex, RACHS-1, STAT, CPB time, cross-clamp time, MUF, ventricular function. |
|  | 14(b) | Indicate number of participants with missing data for each variable of interest. | Methods, Table 1 footnotes. Operative variables available for 329 of 435. |
|  | 14(c) | Cohort: summarize follow-up time. | Not applicable; in-hospital outcomes only. |
| Outcome data | 15 | Cohort: report numbers of outcome events or summary measures over time. | Tables 2–8. All primary and secondary outcomes reported. |
| Main results | 16(a) | Unadjusted and confounder-adjusted estimates with precision (e.g., 95% CI). Indicate which confounders were adjusted for and why included. | Tables 4, 5, 5b, 5c. Forest plot Figure 1. |
|  | 16(b) | Report category boundaries when continuous variables were categorized. | STAT categorization defined per Jacobs 2009 [28]. Hypernatremia thresholds 145 and 148 mmol/L explicit. |
|  | 16(c) | If relevant, translate estimates of relative risk into absolute risk for a meaningful time period. | Results, Clinical Implications. Approximately 1–2 fewer ventilation days per patient; AKI absolute rate 27.7% vs. 41.3%. |
| Other analyses | 17 | Report other analyses done (e.g., subgroups, interactions, sensitivity analyses). | STAT-stratified analysis with interaction test. Hypernatremia analyses (Table 10). Multiple sensitivity analyses (Tables 5, 5b, 5c). |
| **Discussion** |  |  |  |
| Key results | 18 | Summarize key results with reference to study objectives. | Discussion, opening paragraph. Conclusion. |
| Limitations | 19 | Discuss limitations of the study, taking into account sources of potential bias or imprecision. | Discussion, Limitations section (single comprehensive paragraph). |
| Interpretation | 20 | Cautious overall interpretation considering objectives, limitations, multiplicity of analyses, results from similar studies. | Discussion, multiple subsections. Comparison With Prior Literature (Table 9). Magnitude of Effect and Plausibility. |
| Generalizability | 21 | Discuss the generalizability (external validity) of the study results. | Discussion, Limitations. Single-center, community-hospital setting; generalizability to higher-complexity referral centers addressed. |
| **Other information** |  |  |  |
| Funding | 22 | Give the source of funding and the role of the funders for the present study. | Funding statement at end of manuscript. |

*Reference: von Elm E, Altman DG, Egger M, Pocock SJ, Gøtzsche PC, Vandenbroucke JP; STROBE Initiative. The Strengthening the Reporting of Observational Studies in Epidemiology (STROBE) statement: guidelines for reporting observational studies. Ann Intern Med. 2007;147(8):573–577.*
